# From Environmental Burden to Energy Resource: Waste Plastic-Derived Carbons for Sustainable Batteries and Supercapacitors

**DOI:** 10.3390/polym18080983

**Published:** 2026-04-17

**Authors:** Narasimharao Kitchamsetti, Sungwook Mhin, HyukSu Han, Ana L. F. de Barros

**Affiliations:** 1 Department of Microsystems, University of South-Eastern Norway, Campus Vestfold, Raveien 215, 3184 Borre, Norway; 2 Department of Energy and Materials Engineering, Dongguk University, Seoul 04620, Republic of Korea; 3 Division of Materials Science and Engineering, Hanyang University, Seoul 04763, Republic of Korea; 4 Laboratory of Experimental and Applied Physics, Centro Federal de Educação Tecnológica Celso Suckow Da Fonseca, Av. Maracanã Campus 229, Rio de Janeiro 20271-110, Brazil

**Keywords:** waste plastics, carbon materials, energy storage, supercapacitor, rechargeable batteries

## Abstract

The transformation of waste plastics into hydrogen and functional carbon (C) materials represents a promising pathway for achieving both resource recycling and the production of value-added products. Owing to their tunable physicochemical properties, plastic-derived carbons have attracted significant attention in electrochemical energy storage applications. Various C nanostructures, including graphene, porous C, hard C, and C nanotubes (CNTs), can be generated from discarded plastics through thermochemical processes. The electrochemical performance of these materials is closely governed by their structural characteristics, such as pore architecture, specific surface area, heteroatom doping, surface functionalities, and dimensional morphology. This review aims to provide a comprehensive and systematic overview of the conversion of waste plastics into functional C nanomaterials via thermochemical routes, particularly catalytic pyrolysis and carbonization. The resulting C nanostructures are systematically categorized based on their dimensional architectures (0D, 1D, 2D, and 3D) and comparatively analyzed in terms of their structural features and electrochemical performance. Emphasis is placed on the transformation of diverse plastic feedstocks into high-value C materials with tailored dimensional architectures, including graphene, CNTs, C nanospheres, C nanosheets, porous carbons, and their composites. Furthermore, recent progress and critical challenges in utilizing these materials for electrochemical energy storage systems, such as supercapacitors and rechargeable batteries (Li-ion, Na-ion, K-ion, Li-S, and Zn-air), are discussed. Distinct from previous reports, this review highlights the correlation between thermochemical processing strategies, resulting structural features, and electrochemical performance, providing new insights into the rational design of high-performance C materials. These findings are expected to facilitate the advancement of sustainable energy storage technologies while contributing to effective plastic waste valorization.

## 1. Introduction

Plastics, commonly referred to as synthetic polymers, have become indispensable materials in modern society due to their low cost and versatile properties, including high mechanical strength, flexibility, long service life, and resistance to chemical corrosion. Since the mid-20th century, global plastic production has expanded dramatically, from approximately 1.8 million tons in 1950 to 465 million tons in 2018 [1]. Projections suggest that cumulative plastic production could reach 30 billion tons by 2050, accompanied by an estimated 13 billion tons of plastic waste generation [2,3]. Conventional disposal practices for plastic waste primarily involve incineration and landfilling. However, these approaches are neither resource-efficient nor environmentally sustainable. Incineration releases volatile organic compounds (VOCs) and other harmful gaseous emissions, contributing to atmospheric secondary pollution. Meanwhile, landfill accumulation leads to long-term ecological contamination and promotes the formation of microplastics, which pose increasing risks to ecosystems and human health [4]. Against this backdrop, the recycling and valorization of waste plastics have attracted growing research attention as sustainable alternatives for mitigating environmental impact while recovering valuable resources [5].

Achieving a circular plastic economy fundamentally relies on effective recycling strategies, as these approaches preserve the material value of plastics and maintain their inherent physicochemical characteristics within the production cycle. Waste plastic recycling technologies are generally classified into four categories: primary, secondary, tertiary, and quaternary recycling [6,7]. Selection of a specific recycling pathway largely depends on the composition and purity of the plastic waste stream. Primary recycling is applicable to homogeneous and uncontaminated plastic waste, allowing the material to be reprocessed into products with properties comparable to those of virgin polymers. A representative instance is the production of new plastic bottles using blends of recycled and virgin polyethylene terephthalate (PET). This approach is often described as closed-loop recycling because the original material performance is largely preserved [8]. In contrast, secondary recycling, commonly known as mechanical recycling, typically involves mixed or contaminated plastic wastes composed of different polymer types. The recycled materials obtained through this process generally exhibit inferior mechanical and structural properties compared to the original plastics. Therefore, it is characterized as open-loop recycling, as the material undergoes quality degradation during reprocessing [9,10]. Mechanical recycling is one of the most widely adopted approaches for plastic waste management, involving physical reprocessing techniques such as shredding, melting, and remolding. Typical examples include the production of filaments for material extrusion-based additive manufacturing (3D printing) and conventional injection molding processes, which enable the reuse of recycled polymers in high-value applications. Recent studies have demonstrated that recycled fiber-reinforced polymer composites can be successfully processed via material extrusion while maintaining acceptable mechanical properties, highlighting the potential of this approach for sustainable manufacturing [11]. In addition, the reprocessing of waste fibers for thermoplastic composites through mechanical routes has been extensively explored [12]. Tertiary recycling, also referred to as chemical recycling, involves the depolymerization or transformation of plastic waste into monomers, fuels, or other value-added chemicals through processes such as pyrolysis, gasification, and solvolysis. In addition to these conventional approaches, vitrimerization has recently emerged as a promising chemical recycling strategy, particularly for thermoset polymers. Vitrimers are crosslinked polymer networks containing dynamic covalent bonds that can undergo associative exchange reactions, enabling reprocessing and reshaping without complete depolymerization. This approach allows thermoset materials, which are traditionally difficult to recycle, to be reprocessed in a manner similar to thermoplastics, thereby extending their lifecycle and improving sustainability. Recent studies have demonstrated the potential of vitrimer chemistry for the recycling and upcycling of polymeric materials [13,14,15]. Quaternary recycling, in contrast, is generally applied to plastic wastes that are highly contaminated or unsuitable for sorting and material recovery. In this pathway, energy is extracted from plastics through thermal incineration processes [16]. Despite the technical feasibility of these recycling strategies in promoting a circular plastic economy, economic challenges remain significant. In many cases, recycling operations are more costly than the direct manufacture of plastics from fossil-based raw materials, which limits their large-scale industrial adoption.

Over the past decade, chemical upcycling has emerged as a prominent strategy for managing waste plastics. Through this approach, discarded polymers can be converted into value-added products, including C-based materials, fuels, and small molecular compounds [17]. Among various techniques, thermochemical processes are particularly favored due to their operational simplicity, efficiency, and broad applicability [18]. However, since plastic waste streams typically consist of mixed polymers, presorting is generally necessary before chemical recycling. Although this step improves product quality by eliminating contaminants and enabling treatment of complex mixtures [19,20], it inevitably increases processing costs and operational complexity. To overcome these limitations, thermochemical routes, for instance, pyrolysis, which convert mixed plastics into hydrogen and high-value C materials, have attracted growing attention [21,22]. This strategy reduces the need for extensive sorting and offers greater flexibility in feedstock utilization [23]. Over the past few years, significant research efforts have focused on generating hydrogen from waste plastics [24,25]. At the same time, growing interest has been directed toward converting plastic waste into C materials with diverse morphologies and multifunctional applications [26,27,28]. Transforming waste plastics into hydrogen and high-value C products presents an appealing pathway toward a sustainable circular economy. Benefiting from their adjustable physicochemical characteristics, plastic-derived carbons have demonstrated remarkable progress in electrochemical applications.

Several comprehensive reviews have addressed the transformation of plastic wastes to C products [2,28,29,30]. For instance, Wang and co-workers [29] summarized recent progress in plastic-derived carbons for applications in energy storage, environmental remediation, and organic synthesis. Sahoo’s team [30] focused on advances in C materials from plastic waste for supercapacitors (SCs) use. Lee and colleagues [31] reviewed strategies for upcycling plastic waste into high-value C products, with particular emphasis on synthetic approaches. More recently, Ji’s group [2] discussed catalytic routes for transforming plastic waste into advanced C materials. Overall, these reviews primarily concentrate on preparation techniques, while practical applications are generally outlined in a relatively brief manner. Given the accelerating progress in energy storage technologies, waste plastic-derived C nanomaterials (CNMs) are emerging as promising candidates for energy storage systems.

This review systematically summarizes the fabrication of CNMs from waste plastics via numerous thermochemical transformation strategies. It further discusses recent progress in transforming different types of plastic waste into C materials with diverse dimensional architectures, including C dots (CDs), two-dimensional (2D) graphene, C nanotubes (CNTs), C nanospheres (CSs), C nanosheets (CNSs), and three-dimensional (3D) porous carbons (PCs) and their composites. In addition, the latest developments and remaining challenges associated with the application of plastic-derived CNMs in emerging energy storage systems, for instance, SCs and rechargeable batteries, are comprehensively addressed.

## 2. Production of C-Based Materials via Plastic Waste Conversion

### 2.1. C-Based Materials

**Figure 1:**
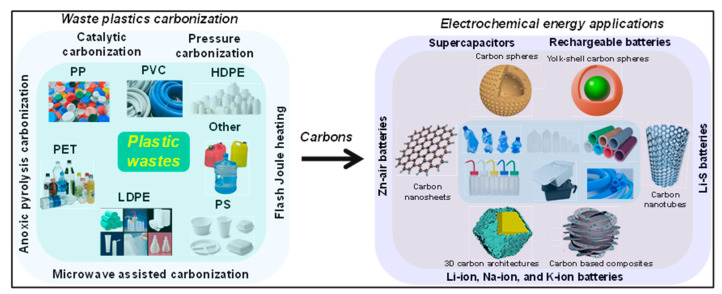
Diagram depicting the transformation of plastic wastes into functional CNMs for energy-related electrochemical devices.

Plastics can typically be divided into three main groups based on their end-use applications. General-purpose plastics, such as high-density polyethylene (HDPE), low-density polyethylene (LDPE), polypropylene (PP), poly (vinyl chloride) (PVC), polystyrene (PS), and PET, are widely used in both consumer goods and industrial components owing to their high production volumes, versatile applications, ease of fabrication, and relatively low cost [29]. In contrast, engineering plastics, including polyamide, polycarbonate, and polyformaldehyde, are mainly utilized in load-bearing or structural components, as they exhibit superior mechanical properties, greater resistance to stress, and improved thermal stability [32,33,34]. Specialty plastics, including fluoropolymers and silicones, are typically reserved for high-performance or niche applications, including the aviation and aerospace sectors. Among these categories, general-purpose plastics account for the largest share of global production and constitute the primary source of plastic-related environmental pollution. Consequently, research efforts have increasingly concentrated on the recycling of commodity plastic waste. Through high-temperature carbonization under controlled conditions, discarded plastics can be transformed into C materials with tailored structures and properties suitable for various electrochemical energy storage applications (Figure 1). Nevertheless, differences in processing routes and operational parameters often result in distinct morphological and structural characteristics [35,36]. The C materials display a wide range of allotropes, extending from 0D to 3D architectures. Among their structural characteristics, pore configuration, specific surface area (SSA), heteroatom incorporation, and surface functionalization are key factors governing their electrochemical behavior.

The transformation of waste plastics into value-added C products generally involves multiple processing steps. The specific routes and operational parameters depend on both the plastic feedstock and the desired final product. Among these steps, carbonization is the central process responsible for forming C structures and is included in virtually all plastic upcycling strategies. Typically, the overall procedure consists of pre-treatment, carbonization, and post-treatment stages [31]. Prior to carbonization, waste plastics are usually subjected to pre-treatment methods, such as mechanical grinding, reducing particle size and enhancing material stability, thereby improving C yield. The C nanomaterials derived from waste plastic carbonization commonly include graphene [37], porous C [2], and CNTs [38]. Graphene possesses a 2D honeycomb lattice with single-atom thickness, which endows it with outstanding electrical conductivity and mechanical robustness. It exhibits remarkable physical properties, including carrier mobility of up to 15,000 cm^2^ V^−1^ s^−1^ at ambient atmosphere, theoretical thermal conductivity approaching 5300 W m^−1^ K^−1^, and a Young’s modulus of approximately 1 TPa [39]. Porous C features a 3D interconnected pore network spanning micro-, meso-, and macro-pores, resulting in high SSA (as high as 3000 m^2^ g^−1^) and excellent adsorption capability. Its surface chemistry can also be tailored through heteroatom incorporation [40]. CNTs are 1D tubular nanostructures consisting of C atoms, known for their greater mechanical strength, low density, superior electrical conductivity, and chemical robustness. Their unique tubular morphology and high aspect ratio enable efficient electron transport and strong axial mechanical performance. The distinct structural characteristics of these nanomaterials dictate their suitability for various electrochemical energy storage and catalytic applications. Further detailed discussions of plastic-derived C nanomaterials are available in the literature [2,31].

In addition to the fundamental mechanisms described above, the physicochemical properties of the resulting C materials are strongly influenced by processing parameters. Among these, temperature plays a crucial role in determining the degree of graphitization, pore structure, and surface area. Lower carbonization temperatures typically yield amorphous carbons with abundant surface functional groups, whereas higher temperatures promote graphitic ordering, enhanced electrical conductivity, and the development of well-defined porous structures [41,42]. The reaction atmosphere also significantly affects the material properties. Inert atmospheres (e.g., N_2_ or Ar) favor C formation and structural stability, while reactive atmospheres (e.g., CO_2_, steam, or NH_3_) can induce activation, leading to increased porosity, surface area, and heteroatom incorporation [43]. For instance, NH_3_ treatment can introduce N functionalities, which enhance electrochemical activity and wettability [44]. In addition, catalysts play a decisive role in tailoring nanostructure morphology and functionality. Transition metal catalysts (e.g., Fe, Ni, Co) facilitate the formation of graphitic structures such as CNTs and graphene via catalytic graphitization mechanisms [45,46]. Catalysts can also promote the formation of hierarchical pore structures and improve the distribution of heteroatoms, thereby enhancing electrochemical performance. Therefore, precise control over processing parameters is essential for tuning the structural features and optimizing the performance of plastic-derived C materials for energy storage applications.

In practical scenarios, waste plastic streams are rarely composed of pure polymers but instead contain a wide range of additives, including plasticizers, flame retardants, stabilizers, pigments, and inorganic fillers, as well as contaminants such as heavy metals and halogenated compounds [47]. During thermochemical conversion, these components undergo complex transformations, including volatilization, decomposition, and partial retention within the C matrix. For instance, halogen-containing additives may release corrosive gases (e.g., HCl, HBr), while metal-based additives can remain as residual impurities, potentially affecting the catalytic processes and structural evolution of C materials [48,49]. These impurities can significantly influence the physicochemical properties of the resulting carbons by altering graphitization behavior, introducing defects, blocking pore structures, and poisoning catalytic active sites [41,42]. Consequently, electrochemical performance, particularly conductivity, capacitance, and cycling stability, may be adversely affected. To address these challenges, recent studies have explored various mitigation strategies, including pre-treatment methods (e.g., dehalogenation and metal removal), in situ capture of harmful species using alkaline sorbents, and the development of impurity-tolerant catalytic systems [43]. In addition, molten salt and templating approaches have shown promise in stabilizing C structures and regulating impurity distribution. Despite these advances, the efficient conversion of real mixed plastic waste into high-performance C materials remains a significant challenge, and further research is required to develop scalable and economically viable solutions [45,46].

### 2.2. Correlation Between Plastic Type and Derived C Microstructure

The microstructure of C materials derived from plastic waste is fundamentally governed by the molecular architecture of the precursor polymer, which dictates its thermal decomposition pathway, intermediate species evolution, and carbonization behavior. Establishing a systematic correlation between plastic type and the resulting C microstructure is therefore essential for guiding the rational design of functional C materials [29]. From a structural perspective, plastic waste can be broadly categorized into four representative groups: (i) aliphatic polyolefins (e.g., PE and PP), (ii) oxygen (O)-containing polymers (e.g., PET and PMMA), (iii) nitrogen (N)-containing polymers (e.g., PU and polyamide), and (iv) halogen-containing polymers (e.g., PVC). Each category exhibits distinct pyrolysis characteristics, leading to markedly different C microstructures. Aliphatic polyolefins, composed of saturated hydrocarbon backbones, typically undergo random chain scission during pyrolysis, generating volatile hydrocarbons with limited formation of stable aromatic intermediates [32,33,34]. As a result, the C yield is generally low, and the obtained C structures are predominantly amorphous or turbostratic with a low degree of graphitization. In contrast, O-containing polymers such as PET possess aromatic rings in their backbone, which facilitate the formation of thermally stable conjugated structures during carbonization. This promotes the development of more ordered C domains with an increased degree of graphitization, although oxygen evolution can also contribute to pore formation. N-containing polymers introduce an additional level of structural complexity. During thermal treatment, N functionalities can be partially retained within the C matrix, resulting in in situ N doping [35,36]. This leads to defect-rich C structures with modified electronic properties and enhanced surface reactivity. Such heteroatom-doped carbons are particularly advantageous for applications in catalysis and energy storage. Meanwhile, halogen-containing polymers such as PVC undergo dehydrochlorination at relatively low temperatures, releasing HCl and generating unsaturated C structures. This process not only promotes crosslinking and aromatization but also contributes to the formation of abundant microporosity and structural defects. Overall, the evolution of C microstructure from plastic precursors follows a general relationship of “polymer structure → decomposition mechanism → intermediate chemistry → C architecture.” Aliphatic polymers tend to yield disordered carbons, whereas aromatic and heteroatom-containing polymers favor the formation of more functionalized and structurally diverse C materials. This structure-property correlation provides a useful framework for predicting C characteristics and tailoring materials through appropriate selection or blending of plastic feedstocks [2,31]. To further clarify the relationship between plastic precursors and C microstructures, a systematic summary of polymer type, decomposition mechanism, and resulting C characteristics is provided in Table 1.

### 2.3. Preparation Approaches

**Figure 2:**
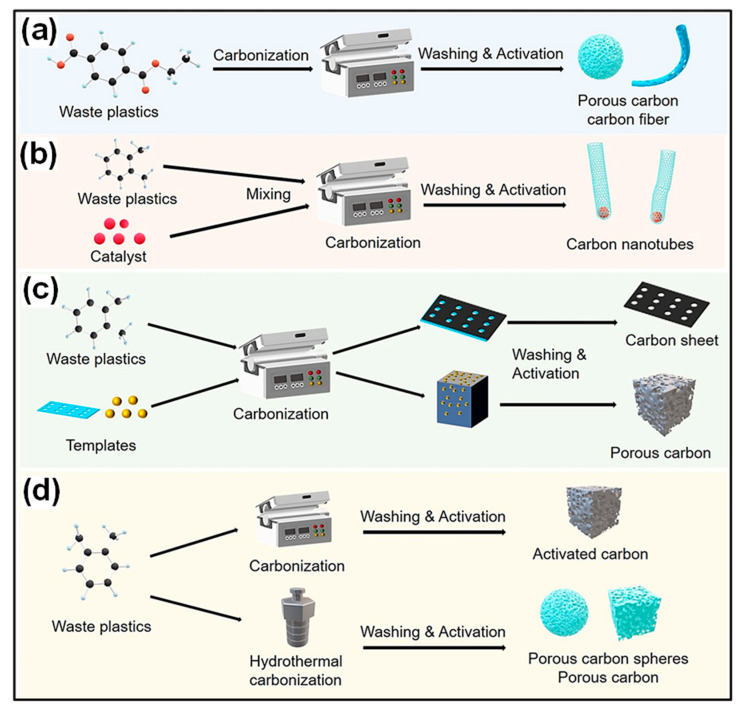
Typical carbonization routes employed to convert waste plastics into C materials. Adapted from [36]. Copyright 2023, Springer Nature.

Waste plastics are commonly converted into carbonaceous products through high-temperature carbonization carried out under controlled atmospheres, with the assistance of catalysts, templates, or elevated pressures. By regulating these processing parameters, CNMs with varied structural features and physicochemical characteristics can be obtained (Figure 2a–d). Thermochemical routes, including inert pyrolysis, catalytic pyrolysis, and pressure-assisted pyrolysis, are widely employed for this carbonization method [36]. Pyrolysis is widely regarded as an effective approach for upgrading waste plastics. During thermal or catalytic pyrolysis, polymers decompose at elevated temperatures (typically 400–600 °C) in an oxygen-free environment, yielding char, condensable liquid products, and non-condensable gases. Incorporation of catalysts may improve both product yield and quality by facilitating reactions including cracking, aromatization, and isomerization, while simultaneously reducing the required reaction temperature and duration [50]. Each preparation route operates under specific conditions and produces C materials with distinct structures. For instance, oxygen-free pyrolysis and hydrothermal carbonization (HTC) typically yield amorphous carbons, whereas coupling pyrolysis with chemical vapor deposition (CVD) promotes the formation of graphitized C structures [51,52,53].

The C materials, including graphene, PCs, and CNTs, have been widely synthesized from plastic waste. For example, single-layer graphene has been produced through an ambient-pressure CVD route using discarded plastics as the C feedstock. In this approach, mixed polyolefins (PP and PE) were initially pyrolyzed at 500 °C to generate hydrocarbon (HC) gases. These gaseous intermediates were subsequently introduced into a CVD reactor containing Copper foil under H_2_/Ar atmospheres at 1020 °C. The flow rate of HC precursors significantly influenced graphene growth. A lower injection rate favored the formation of monolayer graphene with well-defined honeycomb domains, whereas a higher supply rate resulted in multilayer graphene structures [54]. In addition, micrometer-scale multilayer graphene foils were prepared from various plastic wastes (PMMA, PS, PP, PVC, PE, and PET) through a solid-state CVD approach at 1050 °C using Ni foil as the catalytic substrate. The resulting graphene films were self-supporting and mechanically robust, enabling handling without auxiliary substrates. Raman analysis showed comparable I*_D_*/I*_G_* ratios (0.03–0.113) for most precursor-derived samples, while PMMA-based graphene exhibited a higher I*_D_*/I*_G_* value (0.649), suggesting a greater density of structural defects and edge sites [55].

Flash Joule heating (FJH) has recently been developed as a bottom-up strategy for the large-scale production of flash graphene from diverse carbonaceous feedstocks [56]. The graphene obtained through this method typically exhibits a turbostratic configuration, in which graphene layers are randomly oriented and weakly stacked, thereby enabling facile exfoliation into single-layer sheets [56]. During FJH processing, the C precursor is exposed to a high-energy electrical pulse that rapidly elevates the temperature to approximately 2750 °C within <100 ms, inducing instantaneous graphitization [57]. This ultrafast heating step is followed by rapid cooling to room temperature within a few seconds. Concurrently, heteroatomic and non-C volatile species are swiftly expelled during the flashing process [57]. In recent years, Tour and co-workers [56,58] have devoted significant efforts to producing flash graphene from waste plastics via the FJH technique. In particular, they [56] achieved the ultrafast preparation of holey and wrinkled flash graphene (HWFG) from mixed plastic residues within a few seconds by utilizing in situ salt decomposition to create and preserve pore structures during the flashing process. The HWFG produced under high-current conditions possessed a high SSA (874 m^2^ g^−1^) and a hierarchical porous framework comprising micro-, meso-, and macro-pores. Benefiting from these structural characteristics, the material exhibited promising electrocatalytic hydrogen evolution reaction (HER) activity, featuring good durability, low overpotential, and a favorable Tafel slope. In addition, it functioned effectively as an anode for Li-metal batteries, enabling stable cycling at high discharge rates.

Plastic wastes have been widely recognized as viable feedstocks for synthesizing 3D PCs [59]. Owing to their interconnected pore networks and continuous conductive frameworks, these carbons have found extensive applications in electrocatalytic energy conversion and storage systems, where improved electrolyte penetration and accelerated mass transport are essential [60]. The conversion of plastic waste to CNMs generally relies on thermochemical routes performed under diverse conditions, including inert or oxidative environments, with or without catalytic assistance, and under ambient or elevated pressures. Such treatments typically involve pyrolysis and subsequent carbonization, during which volatile HCs evolve and solid C residues are generated. Nevertheless, simple carbonization of plastics often produces carbons with limited porosity [61], making an additional activation process necessary to construct highly porous architectures.

**Figure 3:**
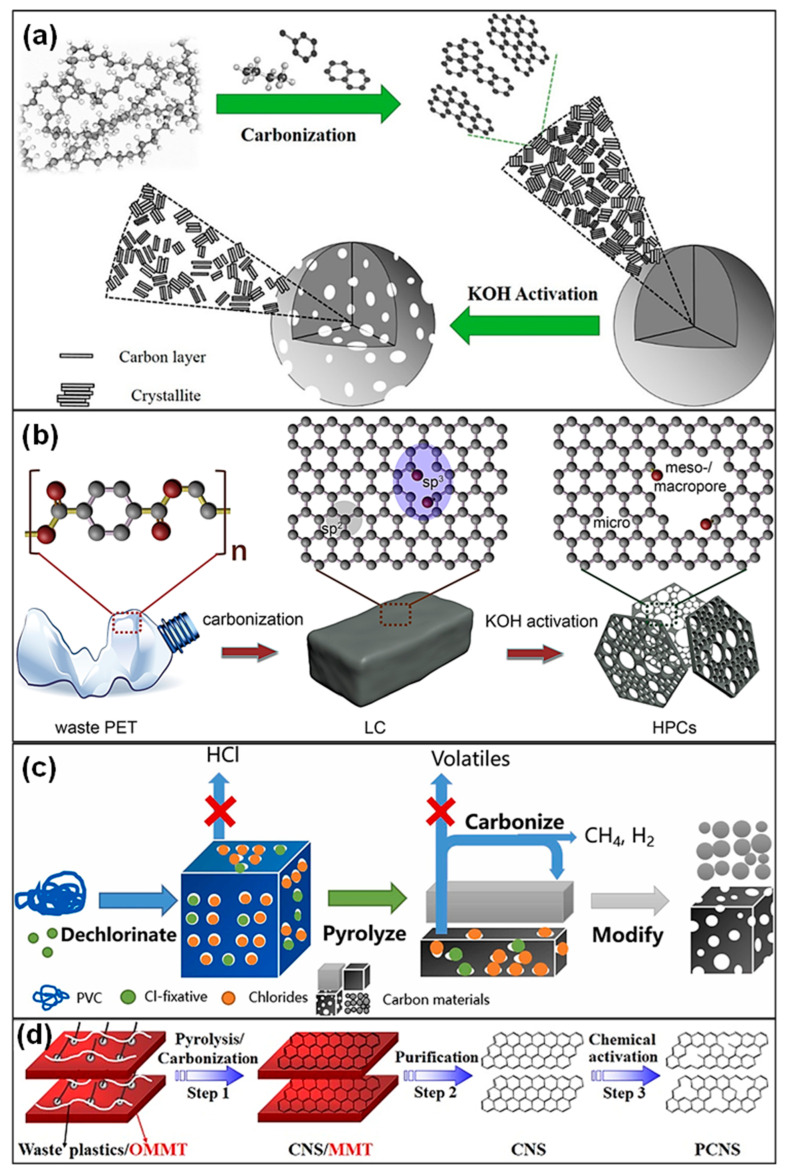
Schematic illustrations showing the preparation routes of porous C materials derived from different plastic precursors: (**a**) LDPE-based porous C produced through autogenic pressure carbonization followed by KOH activation. Adapted from [62]. Copyright 2019, American Chemical Society. (**b**) PET-derived porous C obtained via carbonization and KOH activation. Adapted from [63]. Copyright 2020, Elsevier B.V. (**c**) The C materials prepared from PVC employing a one-step pyrolysis process in a sealed reactor. Adapted from [65]. Copyright 2022, Elsevier B.V. (**d**) Porous C NSs (PCNSs) prepared from mixed plastic waste. Adapted from [68]. Copyright 2019, Elsevier B.V.

For instance, He’s group [62] synthesized hierarchical porous carbons (HPCs) from LDPE via autogenic pressure carbonization combined with subsequent KOH activation. Carbonization in a sealed, catalyst-free reactor produced nearly 45% solid char, and the following chemical activation step created a well-developed hierarchical pore network (Figure 3a). In a related study, Ewa’s team [63] fabricated PET-based HPCs with an ultrahigh SSA of 2238 m^2^ g^−1^ and a meso-/macro-pore volume of 0.51 cm^3^ g^−1^, which was attributed to cooperative etching of sp^2^- and sp^3^-hybridized C domains (Figure 3b). Furthermore, He et al. [64] converted waste PET into methane-rich gas and PC materials through autogenic pressure pyrolysis followed by activation. Compared with ZnCl_2_ treatment, KOH activation produced carbons featuring a more distinct hierarchical pore structure, higher SSA (2683 m^2^ g^−1^), and richer surface functionalities. Zhang and colleagues [65] proposed a one-step strategy to simultaneously convert PVC into PCs, syngas, and chloride compounds. Process comprised sequential dechlorination through chlorine fixation, carbonization of the resulting dechlorinated polyene intermediates, and subsequent activation of the C products (Figure 3c). Chemical reagents like ZnO and KOH were suggested to function dually as activating and chlorine-fixing agents during preparation of porous carbons from halogenated plastics. In addition, alkali and alkaline-earth metal salts have been reported to assist in the pyrolysis of brominated plastic wastes, where alkali pretreatment promotes the effective fixation of bromine (Br) in the char matrix [66,67].

The physicochemical and structural characteristics of porous carbons are strongly dependent on the type of plastic precursor. Generally, polyolefin plastics such as PE and PP tend to form mesoporous honeycomb-like structures during pyrolysis, exhibiting SSAs in the range of 800–2200 m^2^ g^−1^. In contrast, aromatic polymers, including PS and PET, tend to produce carbons with higher degrees of graphitization owing to the rigidity of their aromatic backbones, as evidenced by I*_D_*/I*_G_* values below 0.9. Plastics containing heteroatoms, such as PET and PVC, can introduce surface polarity through residual oxygen or chlorine species (1–3 wt %), although precise temperature regulation during pyrolysis is essential to prevent structural collapse. Overall, the intrinsic chemical composition of the plastic feedstock determines pore structure, defect density, and surface functionality of the derived carbons. Aromatic ring structures influence graphitization degree, whereas heteroatom content determines surface functionality [2]. Consequently, selecting suitable plastic feedstocks based on the intended application (i.e., SCs or batteries) is recommended. Furthermore, the compositional complexity of plastic waste significantly limits the effectiveness of conventional synthesis methods. Therefore, developing universal approaches for producing homogeneous porous carbons from real mixed plastic waste is essential. Tang’s group [68] synthesized porous CNSs (PCNSs) through catalytic carbonization of mixed plastic waste in the presence of organically modified montmorillonite (OMMT), followed by KOH activation (Figure 3d). The OMMT not only promoted the thermal decomposition of plastics but also functioned as a template to direct the in situ formation of CNSs from the degradation intermediates. Subsequent chemical activation generated PCNSs with well-developed hierarchical pore structures.

**Figure 4:**
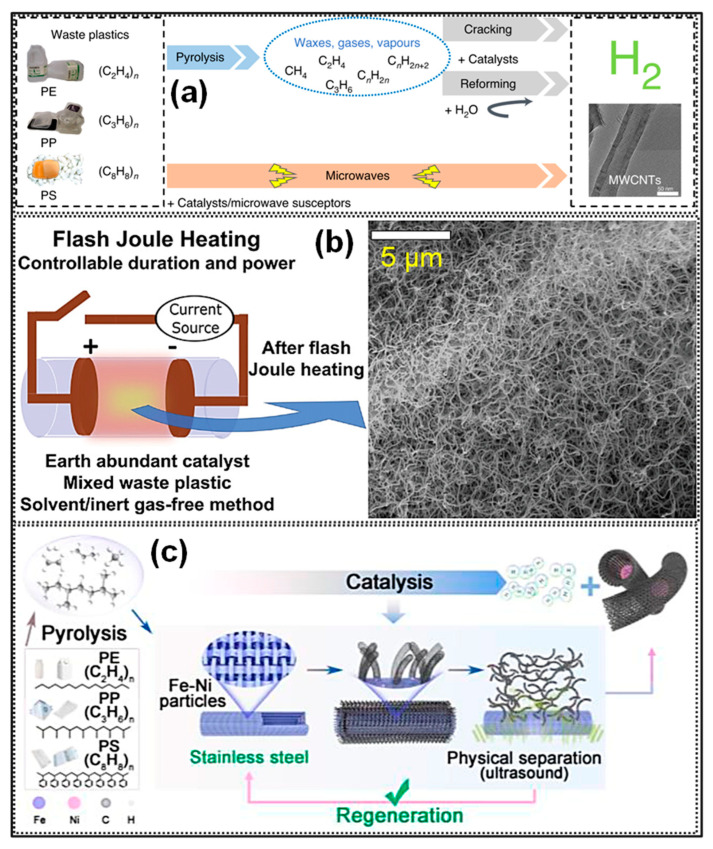
Schematic illustrations of processes for converting waste plastics into hydrogen and CNTs: (**a**) comparison of the two-stage pyrolysis reforming route and the single-stage MW catalytic activity. Adapted from [74]. Copyright 2020, Springer Nature. (**b**) FJH-assisted catalytic conversion of waste plastics to CNTs. Adapted from [75]. Copyright 2023, Wiley-VCH. (**c**) Catalytic pyrolysis over monolithic multilayer stainless-steel mesh catalysts for producing MWCNTs and hydrogen. Adapted from [79]. Copyright 2023, PNAS.

CNTs are 1D tubular materials constructed from sp^2^-hybridized C atoms and are distinguished by their exceptional mechanical strength, lightweight nature, superior electrical conductivity, and chemical robustness [2]. CNTs are generally synthesized via three primary approaches: laser ablation, arc discharge, and CVD [69]. Among these, CVD is most extensively adopted because of its cost-effectiveness, straightforward operation, and high productivity, typically conducted at 600–1200 °C with product yields above 90%. This method has also been extended to the transformation of waste plastics into CNTs, leading to enhanced yield and structural quality [70,71]. With the increasing availability of renewable electricity, electrically driven heating strategies, for instance, microwave pyrolysis [72,73,74] and FJH [75] have gained considerable attention. In particular, microwave-assisted catalytic pyrolysis provides rapid and homogeneous heating, thereby accelerating reaction kinetics and improving catalytic behavior. Peter and co-workers [74] developed a single-step microwave-assisted pyrolysis strategy for HDPE conversion. In this method, HDPE was mechanically blended with FeAlO*_x_* catalyst particles at a 1:1 mass ratio, where the catalyst functioned as an efficient microwave (MW) absorber. Upon MW irradiation, electromagnetic energy was preferentially absorbed by the catalyst particles, inducing localized heating while the surrounding polymer remained initially unheated. The rapid and selective heating facilitated effective polymer decomposition, suppressed undesirable side reactions typically encountered in conventional catalytic pyrolysis, and enabled controlled hydrogen generation with high efficiency. Continuous plastic feeding resulted in a C yield of up to 1560 mg g^−1^ catalyst, with the obtained product comprising over 92% multiwalled CNTs (MWCNTs) (Figure 4a).

Tour’s team [75] employed a FJH strategy to synthesize CNTs from plastic wastes with tunable morphologies (Figure 4b). Diameter and structural features of the CNTs were regulated by adjusting the type and loading amount of transition-metal (TM) catalysts, including Fe, Ni, and Co [76,77,78]. The resulting CNTs exhibit excellent mechanical performance and significantly enhance the tensile strength and toughness of nanocomposites due to their strong reinforcing effects and good interfacial bonding with matrix materials [75]. Plastic-derived CNTs exhibit excellent electrical and thermal conductivities, approaching those of pristine graphene. Furthermore, surface functionalities including -OH and -COOH groups can be incorporated or tailored to enhance interfacial compatibility with various substrates, thereby expanding their applicability in energy storage systems and as catalyst supports [2]. Collectively, these CNMs offer considerable promise for resource valorization, performance enhancement, and cost-effective material production. However, although the simultaneous generation of CNTs and hydrogen from plastic wastes using powdered catalysts is technically viable, the process is limited by relatively low atomic utilization efficiencies, with C recovery below 50% and hydrogen recovery under 60%, in addition to suboptimal product purity. Furthermore, the application of MW and FJH methodologies in plastic pyrolysis faces several challenges. For MW pyrolysis, nonuniform electromagnetic field distribution can cause temperature instability, leading to reduced product purity during industrial scale-up, while equipment fabrication and maintenance costs remain high. In the case of FJH, although extremely high temperatures (~3000 °C within milliseconds) can be achieved, its transition from laboratory research to large-scale industrial application is hindered by limited technological maturity and constraints related to feedstock adaptability.

The recovery of synthesized CNTs typically requires removal of powdered catalyst supports [80]. Conventional purification strategies predominantly involve selective oxidation and chemical treatments, which are energy-intensive and require multiple post-treatment steps [81]. Moreover, such processes may compromise the structural integrity of the supported catalysts, thereby hindering their recyclability. To overcome these limitations, Zhang’s team [79] proposed a pyrolysis-catalytic deconstruction strategy employing a monolithic multilayer stainless-steel mesh as the catalyst to produce MWCNTs and hydrogen (Figure 4c). Prior to reaction, the mesh underwent acid etching followed by air calcination to improve its redox capability and increase surface roughness, thereby enhancing active-site exposure. During the pyrolysis-catalysis process, macromolecular CVD occurred on the modified mesh surface, leading to an elevated C deposition rate. Reaction intermediates, including polycyclic aromatic HCs (PAHs), were transformed into CNTs and hydrogen through a vapor-solid–solid activity, with C atoms supplied via surface and interfacial diffusion. Under optimized circumstances, atomic recovery efficiencies reached 86% for C and 70% for hydrogen.

In addition to recovery challenges, the residual state of metal catalysts within C materials is a critical factor influencing their electrochemical performance and practical applicability. Depending on synthesis conditions and post-treatment processes, TMs such as Fe, Co, and Ni may persist in the C matrix in various forms, including atomically dispersed species, metallic nanoparticles (NPs), and oxidized phases (e.g., metal oxides or carbides) [80]. These residual species can significantly affect the physicochemical properties of the resulting materials. In the context of electrochemical energy storage, particularly in LIBs and SIBs anodes, residual metal species may act as catalytic centers for electrolyte decomposition, leading to unstable SEI formation. This can result in continuous SEI rupture and regeneration, increased irreversible capacity loss, and, under certain conditions, the promotion of dendritic growth, thereby raising safety concerns [82]. While a limited amount of well-dispersed metal species may enhance conductivity or provide additional redox activity, uncontrolled metal residues generally deteriorate long-term cycling stability and reliability. To mitigate these issues, various purification strategies have been developed, including acid leaching, thermal oxidation, and combined chemical-thermal treatments [79]. Acid washing (e.g., using HCl or HNO_3_) is widely employed for removing metal NPs; however, it may introduce structural defects, reduce electrical conductivity, and generate secondary waste streams. Thermal oxidation can selectively remove exposed metal species but may also lead to C loss and pore collapse. Despite these approaches, achieving complete metal removal without compromising structural integrity remains challenging [72,73,74]. Furthermore, these purification steps introduce additional energy consumption, processing complexity, and economic cost, which may limit large-scale implementation. Therefore, developing synthesis strategies that minimize catalyst residue or enable facile catalyst recovery is essential for advancing the practical application of plastic-derived CNTs and related C materials.

### 2.4. Prospects for Future Development

Amid the intensifying global challenge of plastic pollution, the valorization of waste plastics into high-value products has become an important pathway toward sustainable development. The conversion of discarded polymers into advanced C-based materials, including graphene, CNTs, and PCs, facilitates resource recovery while simultaneously providing high-performance materials for renewable energy systems, environmental remediation technologies, and electronic applications. This dual benefit delivers both economic returns and ecological gains. Nevertheless, practical deployment requires overcoming several key obstacles, such as maintaining operational stability, achieving scalable production, and minimizing environmental impacts. In general, widely generated plastic wastes, including PET, PE, PP, and PS, can be converted to 2 major classes of CNMs, namely PCs and CNTs, via different formation mechanisms. During carbonization and activation, polymer chains first experience random cleavage, forming liquid-phase intermediates. When the temperature rises to approximately 500–800 °C, aromatic structures progressively condense via Diels-Alder reactions, leading to the formation of 2D graphite-like microcrystalline domains. The introduction of chemical activating agents, such as KOH or ZnCl_2_, facilitates the development of porous C through synergistic mechanisms: (1) intercalation of metal species into C layers to create micropores (<2 nm), (2) oxidative etching to generate meso-pores (2–50 nm), and (3) volatilization of activating agents at elevated temperatures, resulting in interconnected 3D pore channels. For the synthesis of CNTs, transition metal nanoparticles (NPs) such as Fe, Co, or Ni are typically employed as catalysts. In floating catalyst systems, small HC molecules (e.g., CH_4_ and C_2_H_2_) generated during plastic pyrolysis participate in a dissolution-precipitation mechanism on the catalyst surface. Specifically, C atoms initially dissolve into metal NPs, forming a supersaturated solid solution. Upon reaching supersaturation, C segregates from lattice defect sites and precipitates as sp^2^-hybridized graphitic layers. By precisely regulating catalyst particle size (typically 5–10 nm) and reaction time (10–30 min), selective synthesis of either single-walled CNTs (SWCNTs) or MWCNTs can be achieved. Nevertheless, heteroatom impurities containing sulfur and nitrogen in plastic feedstocks may poison catalytic active sites. Therefore, impurity removal through pretreatment steps or the addition of alkaline promoters such as MgO is often required.

Looking ahead, the valorization of plastic wastes to advanced CNMs is expected to follow several major development trajectories. From a technological perspective, emerging approaches, including catalytic pyrolysis and MW-assisted carbonization, are anticipated to enhance conversion efficiency, improve material performance, and lower production costs. In terms of application, these C materials are likely to find expanded use in SCs, rechargeable batteries, catalyst supports, and related areas, thereby contributing to the advancement of sustainable energy systems. At the policy level, governments worldwide are expected to reinforce regulatory frameworks for plastic recycling and incentivize corporate investment in high-value plastic upcycling technologies, ultimately fostering an integrated industrial chain encompassing recycling, conversion, and application. With continued process optimization, large-scale industrial production of C materials derived from waste plastics is expected to become feasible, positioning this technology as a pivotal component of the circular economy.

## 3. Plastic Waste-Derived Carbons for Supercapacitors

The upcycling of waste plastics into electroactive C materials for energy storage applications offers an effective strategy to alleviate plastic pollution [83,84]. At the same time, this approach supports sustainable energy development by fostering circular resource utilization and decreasing reliance on fossil-based feedstocks [85,86,87]. SCs have been widely utilized in contemporary energy storage technologies because of their high power output, outstanding cycling durability, prolonged lifespan, fast charging-discharging characteristics, and broad operating voltage range [88,89]. Their energy storage behavior is primarily governed by two mechanisms: electrostatic double-layer capacitance and pseudocapacitive redox reactions. Based on these mechanisms, SCs are generally classified into electrochemical double-layer capacitors (EDLCs), pseudocapacitors (PCs), and hybrid supercapacitors (HSCs). EDLCs store charge via the physical accumulation of electrolyte ions at the electrode-electrolyte interface, with C-based materials typically serving as the electrode components. In contrast, PCs depend on fast and reversible faradaic reactions occurring on electrode samples such as TM oxides or metal-modified carbons. HSCs integrate both mechanisms by pairing a pseudocapacitive electrode with a double-layer-type electrode. Thus, independent of device configuration, the intrinsic characteristics of electrode materials fundamentally govern the electrochemical properties of SCs [90]. Key physicochemical parameters, including porosity, SSA, chemical composition, and morphology, strongly influence device behavior [91]. Among the available materials, C-based electrodes have been most widely adopted in SC systems. Prior investigations have shown that introducing heteroatoms such as N, O, and S into plastic-derived carbons can markedly enhance electrical conductivity, interfacial wettability, and pseudocapacitive effects, leading to improved electrochemical performance [92]. This section reviews recent progress in utilizing plastic waste derived C products as SC electrodes, with particular focus on clarifying the relationship between C structure and capacitive behavior, thereby highlighting the importance of rational design of high-performance electroactive carbons.

The electrochemical performance of plastic-derived C materials is intrinsically governed by their structural characteristics, including pore architecture, graphitization degree, and surface chemistry. Specifically, a high SSA, particularly dominated by micropores (<2 nm), contributes significantly to electric double-layer capacitance by providing abundant ion adsorption sites [83,84]. However, excessive microporosity may hinder ion transport, especially at high charge–discharge rates. In contrast, meso-pores (2–50 nm) serve as ion buffering reservoirs and transport channels, thereby enhancing rate capability and power density. The degree of graphitization also plays a critical role in determining electrical conductivity. Carbons with higher graphitic ordering facilitate faster electron transport, which is beneficial for both SCs and battery electrodes [91]. Meanwhile, heteroatom doping, commonly observed in carbons derived from N- or O-containing plastics, introduces additional active sites and can contribute to pseudocapacitive behavior, further improving capacitance and energy density. Importantly, the choice of plastic precursor strongly influences these structural features. For instance, polyolefin-derived carbons typically exhibit low graphitization and limited functionality, resulting in moderate electrochemical performance. In contrast, PET-derived carbons can develop more ordered structures, while N-containing polymers yield heteroatom-doped carbons with enhanced electrochemical activity [74]. Therefore, tailoring the precursor composition and processing conditions provides an effective strategy to optimize the structure-performance relationship of plastic-derived carbons.

### 3.1. Porous Carbons

The CNMs derived from waste plastics, including graphene, CNSs, CNTs, HPCs, and C-based heterostructures, have been widely investigated as SC electrode materials because of their tunable structural and compositional features. Representative preparation strategies and applications of these materials in SC systems are compiled in Table 2. Among these candidates, activated carbons (ACs) remain the most commonly employed electrodes owing to their large SSA (generally exceeding 1000 m^2^ g^−1^), substantial pore volume, low cost, and suitable electrical conductivity. As a result, ACs can deliver specific capacitances ranging from 115 to 340 F g^−1^ in both aqueous and organic electrolytes. The physicochemical characteristics are strongly influenced by the nature of the C precursors and the synthesis procedures, including carbonization and activation. These factors play a decisive role in determining the SSA, pore architecture, and pore size distribution of the resulting materials. Increasing the SSA, within a reasonable range, has been recognized as an effective approach to enhance the capacitance of C-based electrodes [64]. To obtain high SSA and large pore volumes, both physical and chemical activation strategies are widely employed. It has been reported that different chemical activating agents, such as ZnCl_2_ and KOH, exert distinct effects on C materials. Activation with ZnCl_2_ generally facilitates the development of graphitic domains, accompanied by a decrease in surface organic functional groups. By comparison, KOH activation typically suppresses graphitization but promotes the formation of a hierarchical pore network with high SSA and rich surface functionalities.

The AC treated with KOH exhibited enhanced electrochemical behavior relative to that activated with ZnCl_2_, which can be attributed to its more favorable structural features. When employed as a SC electrode, the KOH-activated AC delivered a high specific capacitance of 325 F g^−1^ at 0.5 A g^−1^, along with low equivalent series resistance (ESR) and excellent cycle durability [64]. HPCs have been extensively explored as cathodes for SCs due to their favorable pore architecture. In cases where halogen-containing plastics such as PVC are used as C templates, a dehalogenation step is typically necessary prior to carbonization [93]. Additionally, C materials with a higher degree of graphitization or graphene-like structures are known to significantly enhance capacitance retention and long-term cycling stability [94]. Li and co-workers [95] demonstrated the fabrication of 3D graphene from waste tires through a KOH-assisted one-stage pyrolysis strategy. Elevating carbonization temperature from the traditional 800 °C to temperatures exceeding 1000 °C led to the formation of K-vapor, which facilitated C atom rearrangement and promoted the conversion of soft C fractions into graphene-like domains. Throughout this transformation, the morphology progressed from amorphous CSs to bulk C blocks, followed by wrinkled graphene sheets, and ultimately vertically oriented 3D graphene frameworks. The obtained material exhibited high conductivity of 18.2 S cm^−1^, nearly two orders of magnitude higher than that of typical ACs. Benefiting from its interconnected porous network and excellent conductivity, the 3D graphene delivered remarkable capacitive behavior, including superior rate capability and long-term cycling stability, retaining 95.9% of its initial capacitance after 10,000 cycles in 6 M KOH electrolyte when applied as an EDLC electrode. Matranga’s team [94] achieved the transformation of linear LDPE into turbostratic graphene using a KCl/K_2_CO_3_ molten salt system, involving thermal oxidation pretreatment, subsequent carbonization, and catalytic graphitization. The obtained graphene possessed a high SSA of 1800 m^2^ g^−1^ and a total pore volume of 1.16 cm^3^ g^−1^, forming a well-developed hierarchical porous structure. Benefiting from these structural features, the material exhibited excellent electrochemical performance in SCs, delivering a gravimetric capacitance of 175 F g^−1^, an areal capacitance of 3.5 F cm^−2^, and an energy density of 9.45 Wh kg^−1^. Remarkably, even at a high mass loading of 20 mg cm^−2^, the electrode retained 95.8% of its initial capacitance after 100,000 charge–discharge cycles, surpassing the durability requirements of commercial devices.

**Table 2:** Representative studies on CNMs derived from plastic wastes for SCs.

Plastics	Preparation Route	C Material	SC Configuration (Electrolyte)	Specific Capacitance (F g^−1^) [Current Density (A g^−1^)]	Capacitance Retention (%) [Cycle Number]	Ref.
LDPE	Autogenous-pressure carbonization combined with subsequent KOH activation	HPC spheres	Three electrode (6 M KOH)	355 [0.2]	-	[62]
PET		HPCs	EDLCs (6 M KOH)	325 [0.5]	91.8 [5000]	[64]
PET	Carbonization with subsequent KOH activation	HPCs	Three electrode (6 M KOH)	413 [0.5]	-	[63]
PP	Carbonization conducted with ferrocene and S under autoclave conditions	PCNSs		349 [0.5]	99 [10,000]	[96]
Mixed plastics	OMMT-assisted catalytic carbonization followed by KOH activation	HPC NSs	Three electrode (6 M KOH)	207 [0.2]	-	[68]
			Three-electrode (1 M Na_2_SO_4_)	137 [0.2]		
PVC	Ball milling followed by KOH-assisted carbonization	HPCs	Three electrode (6 M KOH)	399 [1.0]	92 [1000]	[97]
PVC	Single-step carbonization in the presence of CaCO_3_, K_2_CO_3_, and melamine	N-doped HPC NSs		347 [0.5]	99.2 [5000]	[93]
Tires	KOH-assisted catalytic pyrolysis	3D graphene	EDLCs (6 M KOH)	324.9 [0.2]	95.9 [10,000]	[95]
Mixed plastics	ZnO- and MMT-assisted catalytic pyrolysis	Graphene NSs	EDLCs (1 M H_3_PO_4_)	377.4 [1.0]	89 [5000]	[98]
Waste mask (PP)	NaOH activation after sulfonation treatment	S-doped porous C	HSCs (6 M KOH)	338.1 [1.0]	98.8 [10,000]	[99]
Waste mask (PP)	Thiourea-assisted molten salt carbonization	N, S co-doped porous C	Three-electrode (6 M KOH)	345.6 [1.0]	93.3 [20,000]	[100]
Waste mask (PP)	Carbonization with subsequent KOH activation	S-doped porous C		651.1 [0.1]	98 [50,000]	[101]
Waste mask	Sulfidation and carbonization	S-doped CNFs	Three-electrode (2 M KOH)	234 [0.5]	96.8 [15,000]	[102]
PP	CuCl_2_-assisted molten-state carbonization	PCNSs	EDLCs (6M KOH)	157 [0.1]	87 [10,000]	[103]
Polyamide	K_2_CO_3_-assisted pyrolysis and activation	Porous C	Two-electrode (1 M H_2_SO_4_)	220 [1.0]	95 [30,000]	[104]
Polyurethane	MgO-templated co-carbonization with subsequent KOH activation	HPCs		310 [0.5]	-	[105]
Polyurethane foam	KOH-assisted pyrolysis and activation	N-doped HPCs	Three-electrode (6 M KOH)	342 [0.5]	87.4 [10,000]	[106]
Polystyrene	Fe_2_O_3_-assisted catalytic pyrolysis with subsequent KOH activation	HPCs		284.1 [0.5]	86.5 [10,000]	[107]
LLDPE	KCl/K_2_CO_3_-assisted pre-oxidation and carbonization	Turbostratic graphene	Two-electrode (1 M H_2_SO_4_)	175 [0.25]	95.8 [100,000]	[94]
Polyolefin (PE, PP, PS)	ZnCl_2_- and melamine-assisted pre-oxidation and carbonization	N-doped HPCs	Two-electrode (2 M KOH)	224.8 [1.0]	91.2 [50,000]	[108]
PP	KOH-assisted sulfidation and carbonization	Carbon nanofiber	Three-electrode (6 M KOH)	194 [0.5]	80.4 [6000]	[109]
PET	KOH-assisted catalytic carbonization	PCNSs		169 [0.2]	90.6 [5000]	[110]
PET	Urea-assisted carbonization after solvothermal treatment with Al cans	N-doped HPCs		355 [0.5]	88.2 [10,000]	[111]
PET	MgO-templated carbonization with subsequent KOH activation	HPCs	Two-electrode (2 M KOH)	191.4 [0.5]	98.2 [5000]	[112]
PET	K_2_CO_3_-assisted one-step pyrolysis	HPCs		332.3 [0.5]	95.9 [10,000]	[113]
PET	Carbonization after solvothermal treatment	NiO_x_@N-doped porous C	Three-electrode (6 M KOH)	581.3 [5 mV s^−1^]	-	[114]
PET	Carbonization after hydrothermal treatment	N-doped mesoporous C		295 [0.5]	98 [400]	[115]
PET	Hydrothermal preparation	Intercalated 0D C dots and 2D CNSs	DELC, (6 M KOH)	237 [1.0]	98 [12,000]	[116]

**Figure 5:**
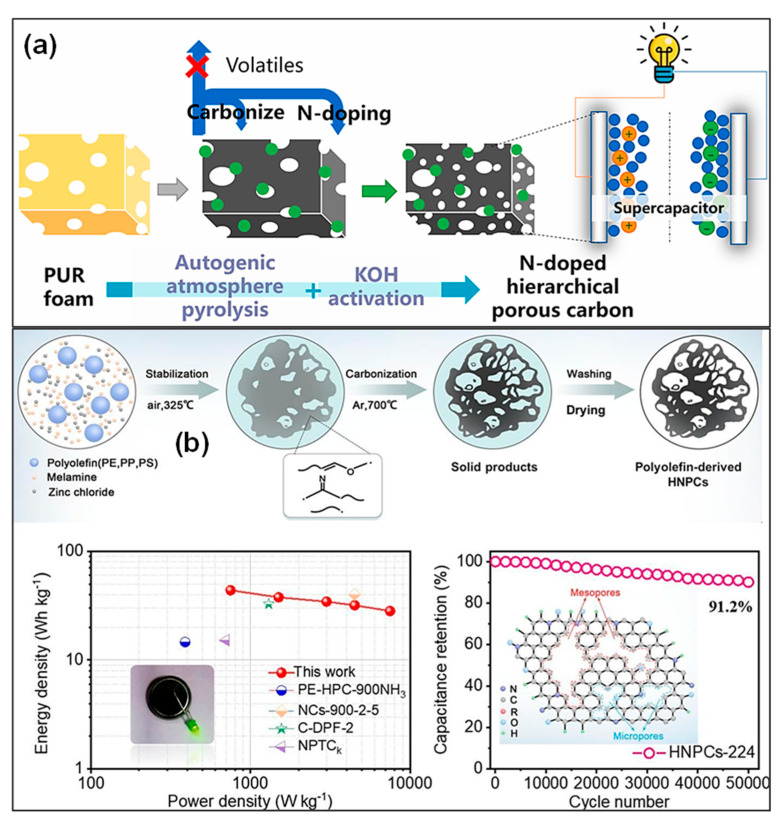
(**a**) Preparation of HNPCs from PUR foam for SC applications. Adapted from [107]. Copyright 2020, Elsevier B.V. (**b**) Fabrication of HNPCs from polyolefin waste for SCs. Adapted from [108]. Copyright 2025, Elsevier B.V.

The incorporation of heteroatoms, particularly nitrogen and sulfur, has been widely recognized as an effective approach to improve the electrochemical properties of waste plastic-derived HPCs for SC applications [99,100,102]. In the case of nitrogen doping, two primary strategies are commonly employed: intrinsic doping via nitrogen-rich plastic precursors [106] and extrinsic doping through the addition of nitrogen-containing compounds such as melamine [108] or urea [111]. As illustrated in Figure 5a, nitrogen self-doped HPCs were derived from polyurethane (PUR) through a combined pyrolysis and KOH activation. Additionally, hierarchical nitrogen-doped PCs (HNPCs) were fabricated from polyolefin waste through pre-oxidation followed by ZnCl_2_-assisted carbonization in the presence of melamine as an additional nitrogen precursor. The cooperative interaction between ZnCl_2_ and melamine facilitated the construction of thermally stable C frameworks, leading to a relatively high C yield (34.3%) and nitrogen content (11.2 at. %). Meanwhile, numerous micro- and meso-pores were generated, affording a SSA of 1031.7 m^2^ g^−1^. The HNPC electrode delivered a specific capacitance of 224.8 F g^−1^ at 1 A g^−1^ and retained 91.2% of its initial capacitance after 50,000 charge–discharge cycles (Figure 5b). Furthermore, the corresponding symmetric SCs achieved an energy density of 43.8 Wh kg^−1^ at a power density of 750 W kg^−1^, with 87% capacitance retention over 10,000 cycles in TEABF_4_/AN electrolyte. Li and co-workers [111] reported the transformation of waste PET bottles and aluminum cans into the MIL-53(Al) through a solvothermal synthesis route. HNPCs were subsequently obtained from MIL-53(Al) through carbonization with urea. When employed as SC electrodes, these HNPCs achieved a specific capacitance of 355 F g^−1^ at 0.5 A g^−1^ in a three-electrode system and delivered an energy density of 20.1 Wh kg^−1^ at a power density of 225 W kg^−1^, while retaining 88.2% of the initial capacitance after 10,000 cycles in a two-electrode configuration.

### 3.2. Low-Dimensional Carbons

**Figure 6:**
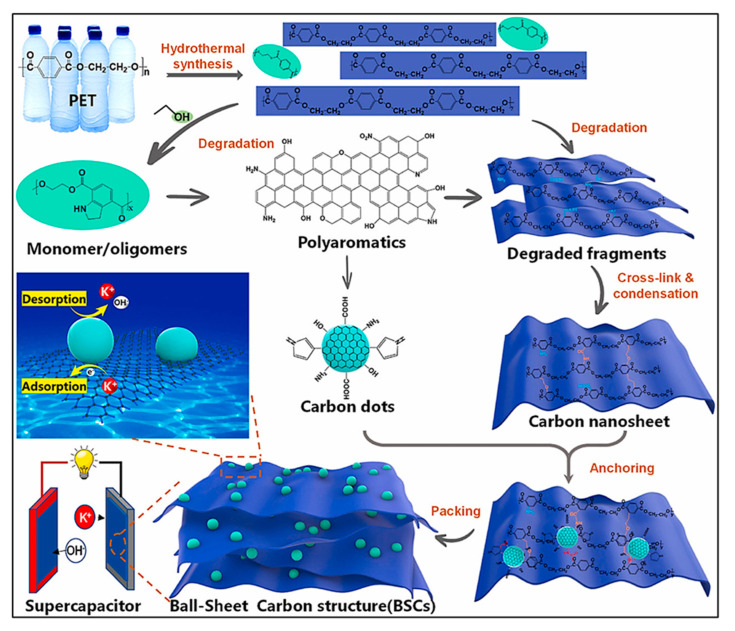
Preparation route of PET-derived ball-sheet C architecture for application in SCs. Adapted from [116]. Copyright 2024, Elsevier B.V.

Three-dimensional porous carbons (i.e., ACs) produced via physical, chemical, or combined activation of waste plastics typically exhibit high SSAs and abundant ion-accessible sites; however, their intrinsic electrical conductivity is often moderate [113]. In contrast, low-dimensional carbons, for instance, 1D CNFs and 2D graphene, form interconnected conductive frameworks with superior electrical transport properties, though they generally suffer from limited SSA and higher production cost or lower scalability. 0D C NPs, including C dots (CDs), offer adjustable SSA owing to their controllable size and morphology. However, their isolated particle nature necessitates the incorporation of conductive, electrochemically inactive additives or other C frameworks to construct functional electrodes [116]. Therefore, integrating multiple C architectures represents a promising strategy to synergistically combine high SSA, enhanced conductivity, and robust cycle stability for advanced energy storage applications. In the study by Wang’s team [116], waste PET was simultaneously transformed into CDs and conductive CNSs through a hydrothermal process. The CDs were uniformly embedded within the CNSs matrix, forming an interconnected ball-sheet C framework. Assisted by HNO_3_ and C_2_H_5_OH, depolymerized PET species underwent condensation to generate CDs featuring benzene-rich cores and surface functional groups such as -OH, -COOH, -NH-, and O=C-NH- moieties. The resulting hybrid C architecture demonstrated high conductivity and substantial ion storage capability when fabricated into SC electrodes (Figure 6). The electrodes delivered specific capacitances of 237 and 198 F g^−1^ at current densities of 1 and 2 A g^−1^, respectively, with only gradual decay at higher rates. Moreover, excellent cycling durability was observed, retaining 98% of the initial capacitance after 12,000 charge–discharge cycles.

Generally, waste plastics can be converted into C materials through two main pyrolysis molding approaches: a single-furnace one-pot process or a dual-temperature zone system [117]. In the one-pot configuration, plastic decomposition and C growth take place simultaneously in the same heating chamber, which tends to generate a significant number of heavy HCs (>C_5_). Such products are unfavorable for CNT formation. Additionally, impurities present in waste plastics (i.e., inorganic fillers) can markedly reduce CNT purity and complicate their separation. In contrast, the dual-temperature system spatially separates the two reactions. Plastics are first pyrolyzed at a relatively lower temperature in the initial zone, gradually producing lighter HCs (C_1_–C_5_) that are more suitable C sources for CVD growth of CNTs. These volatile species are then transported by a carrier gas to a second, higher-temperature zone where carbonization occurs. By independently optimizing the temperatures of pyrolysis and C growth, the dual-zone strategy enables a continuous process and promotes the formation of good-quality CNTs from waste plastics [118].

**Figure 7:**
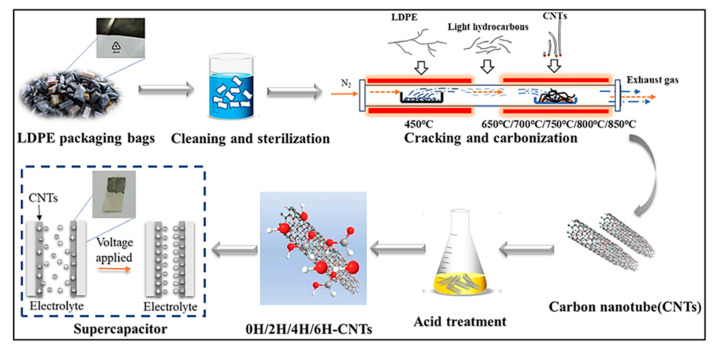
Schematic illustration of the pyrolysis-CVD process for producing CNTs from waste LDPE and their application as SC electrodes. Adapted from [118]. Copyright 2023, Elsevier B.V.

Plastic waste-derived CNTs and their heterostructures have emerged as promising alternative electrodes for SCs owing to their unique pore architectures and high SSAs, which favor enhanced capacitance and prolonged cycling stability [118,119]. Zhang and co-workers [118] synthesized CNTs from waste LDPE using a dual-temperature reaction arrangement for SC applications (Figure 7). At a carbonization temperature of 750 °C, maximum CNT yield (41.9%) and C conversion efficiency (61.2%) were obtained, and the resulting CNTs exhibited well-defined structures. The assembled CNT electrodes retained 93.16% of their initial capacitance and maintained a Coulombic efficiency (CE) of 92.85% after 10,000 charge–discharge cycles. Overall, pyrolysis-based strategies are advantageous due to their operational simplicity and compatibility with mixed plastic feedstocks, enabling the production of C materials with diverse dimensionalities (0D, 1D, 2D, and 3D). These features highlight the potential of pyrolysis for cost-effective upcycling of plastic waste into high-performance C electrodes. Nevertheless, further studies are required to address the recovery and recycling of C materials from end-of-life SCs [120].

### 3.3. Prospects for Future Development

The transformation of plastic wastes into advanced CNMs for SC electrodes has emerged as a prominent research direction at the intersection of resource recovery and electrochemical energy storage. Future investigations are expected to concentrate on performance enhancement, environmentally benign processing, and scalable industrial implementation. From a structural and electrochemical perspective, advanced pore engineering strategies are required to construct hierarchical micro-/meso-/macro-porous architectures, targeting SSAs above 2000 m^2^ g^−1^ and capacitance values exceeding 450 F g^−1^. Establishing a comprehensive database correlating plastic feedstock composition with C material performance will further enable machine-learning-assisted optimization of heteroatom incorporation (i.e., N, S, P), aiming to raise the pseudocapacitive contribution beyond 30%. In terms of sustainable processing, the development of low-temperature (<600 °C) catalytic pyrolysis technologies is crucial to reduce energy consumption by approximately 40% while increasing C yields to over 35% [82,121,122]. In addition, the development of in situ dehalogenation technologies, such as dichlorination for halogen-containing plastics (i.e., PVC) is essential. Achieving a chlorine removal efficiency above 99.9% would effectively prevent catalyst deactivation during the conversion process. From an industrialization perspective, constructing continuous production lines with an annual capacity of 10,000 tons is necessary to lower the manufacturing cost of C materials. To advance long-term sustainability, it is essential to implement a comprehensive life cycle assessment (LCA) framework capable of achieving an estimated reduction of approximately 2.8 tons of CO_2_ emissions per ton of treated plastic. In parallel, unified classification standards for plastic waste, along with performance evaluation and certification systems for derived CNMs, should be established. Realization of these goals requires synergistic collaboration among academic institutions, industry stakeholders, and research organizations, particularly to address challenges in catalyst engineering and scale-up equipment development. Such coordinated efforts will facilitate the conversion of waste plastic from the environmental burden into a strategic precursor.

To better evaluate the practical applicability of plastic-derived C materials, it is important to compare their electrochemical performance with that of commercially available and biomass-derived carbons. Commercial SCs typically employ activated C electrodes with a specific capacitance in the range of ~80–120 F g^−1^ and energy densities of approximately 4–5 Wh kg^−1^ [123]. In contrast, biomass-derived carbons, owing to their hierarchical porosity and tunable surface functionalities, often exhibit significantly enhanced capacitance values, commonly ranging from ~200 to 400 F g^−1^, with some optimized systems exceeding 400 F g^−1^ [124]. Plastic-derived C materials reported in recent studies demonstrate competitive or even superior performance, particularly when engineered with controlled porosity, heteroatom doping, and nanostructured architectures. These materials typically achieve capacitance values comparable to or higher than commercial activated carbons, and in some cases approach those of advanced biomass-derived carbons. However, their performance is highly dependent on processing conditions and precursor composition. Therefore, while plastic-derived carbons show strong potential as sustainable electrode materials, further optimization and standardization are required to consistently match or surpass the performance of state-of-the-art biomass-derived and commercial C materials.

## 4. Rechargeable Batteries

Rechargeable battery technologies, such as LIBs and SIBs, are extensively employed as efficient energy storage systems in contemporary portable electronics, including smartphones and laptop computers, owing to their lightweight characteristics and high energy densities [125,126,127]. The performance of these devices is fundamentally governed by the intrinsic properties of electrode materials, which directly influence energy density and cycling durability. To meet the increasing demand for large-scale energy storage applications, the development of cost-effective electrode materials with rapid charge–discharge capability, high specific capacity, and extended lifespan has become imperative. The CNMs derived from waste plastics have attracted growing attention in battery research owing to their low fabrication cost, scalability, and tunable structural characteristics, which enable their application as both anode and cathode candidates across diverse battery chemistries [29,128]. Particularly, ACs, graphene-like turbostratic carbons, hard carbons, and CNTs synthesized from plastic waste have been widely investigated as anode materials for alkali-ion batteries, demonstrating excellent reversible storage toward alkali metal ions such as Na^+^, Li^+^, and K^+^ [129,130,131]. In addition, these materials have demonstrated promising performance as cathodes in lithium-sulfur (Li-S) and zinc-air (Zn-air) battery systems [132,133].

### 4.1. Advanced Anode Architectures for Alkali-Metal Ion Batteries

#### 4.1.1. Lithium-Ion Batteries

C-based materials are widely regarded as leading candidates for Li^+^ storage anodes due to their exceptional structural stability, natural abundance, and cost-effectiveness. Converting waste plastics into value-added carbonaceous materials offers a sustainable and economically viable strategy for fabricating LIB electrodes [134,135]. A summary of recent studies on waste plastic-derived carbons as LIB anodes is presented in Table 3. Common waste polymers, including PE, PP, and PS, have been widely utilized as C sources. Various controllable carbonization strategies, including catalytic and template-assisted carbonization coupled with activation treatments, have been applied to regulate the morphology, pore structure, and surface functionality of plastic-derived carbons, thereby significantly affecting their electrochemical behavior [136,137,138]. As LIB anodes, porous carbons typically deliver high reversible capacity and outstanding cycling stability, mainly due to shortened Li^+^ diffusion pathways and enlarged electrode/electrolyte interfacial areas that facilitate charge transfer. Moreover, surface functionalization or heteroatom incorporation has proven to be an effective approach for further enhancing electrochemical behavior.

Chemical incorporation of carbonaceous electrodes is an effective strategy to enlarge SSA, introduce additional active binding channels, and enhance both ionic and electronic conductivity, thereby improving Li^+^ storage capability and transport kinetics [138]. Particularly, nitrogen-doped carbons have been extensively investigated as LIB anode materials. For instance, Xu’s group [138] prepared nitrogen-doped porous carbons (NPCs) by carbonizing PS foam in the presence of urea for Li+ storage applications. The optimized sample (NPC-5) possessed a high SSA and a well-developed interconnected porous framework, which facilitated electronic conduction and stable Li^+^ adsorption. As a result, NPC-5 delivered a reversible capacity of 600 mAh g^−1^ after 200 cycles at 1 A g^−1^ and maintained 443 mAh g^−1^ at 5 A g^−1^. The enhanced performance was attributed to its hierarchical pore structure, uniform pore distribution, and nitrogen incorporation, which shortened Li^+^ diffusion pathways and buffered volume variations during lithiation/delithiation.

**Figure 8:**
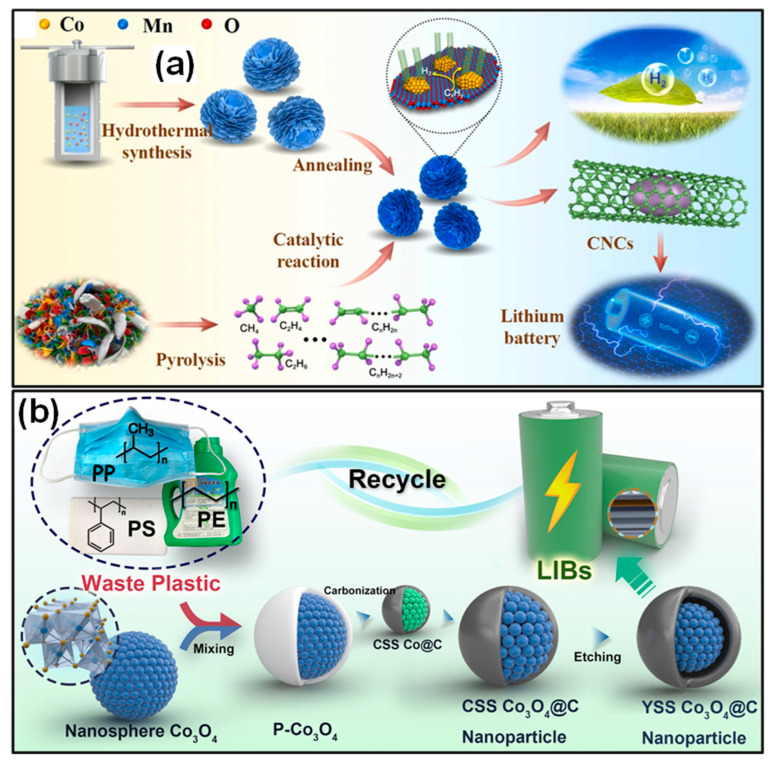
Pictorial illustrations of (**a**) waste plastic upcycling using Co*_x_*Mn_3−*x*_O_4_ spinel catalysts to produce CNCs for LIB anodes. Adapted from [164]. Copyright 2023, Elsevier B.V. (**b**) Conversion of plastic wastes to Co_3_O_4_@C structures for LIB anodes. Adapted from [145]. Copyright 2023, American Chemical Society.

Catalytic pyrolysis/carbonization has been widely recognized as a highly promising strategy for the upcycling of waste plastics, as it enables the simultaneous generation of valuable gaseous products (such as hydrogen) and C materials, including CSs and CNCs [51]. These CNCs can serve as potential anodes for LIBs. For instance, Anke and co-workers [164] designed multiscale-engineered 3D Co*_x_*Mn_3−*x*_O_4_ spinels with precisely controlled composition and abundant active channels, functioning as efficient pre-catalysts for plastic waste conversion. The catalyst microstructure was tailored at the molecular level through hydrothermal preparation, while a 3D rose-type morphology was constructed to create a high density of active sites (Figure 8a). With a pre-catalyst-to-plastic mass ratio of 1:14, CNCs and hydrogen yields reached 41 wt % and 36 mmol g*_pla_.*^−1^, respectively, while the specific yields were 7.48 g*_cat_*^−1^ and 634 mmol g*_pla_.*^−1^g*_cat_*^−1^. When used as LIB anodes, the resulting CNCs demonstrated excellent electrochemical performance, delivering an initial discharge capacity of 770.1 mAh g^−1^ and maintaining a reversible capacity of 522.4 mAh g^−1^ after 100 cycles.

Additionally, Chen’s group [145] reported a high-efficiency strategy for transforming mixed waste plastics (PP, PE, and PS) to nanoscale yolk–shell Co_3_O_4_@C composites for application as LIB anodes. The synthesis involved catalytic carbonization, followed by controlled partial etching of Co_3_O_4_, which served simultaneously as a carbonization catalyst and a Li^+^ storage medium (Figure 8b). The partial etching generated internal void space between the Co_3_O_4_ core and the outer C shell, which effectively accommodated volume changes during repeated lithiation/delithiation cycles, thereby improving electrochemical stability. As a result, these structural characteristics enhanced electrode kinetics and enabled greatly reversible reactions during long-term cycling. The resulting Co_3_O_4_@C delivered capacity of 1066 mAh g^−1^ at 0.1 A g^−1^ over 100 cycles, along with outstanding cycle behavior.

**Figure 9:**
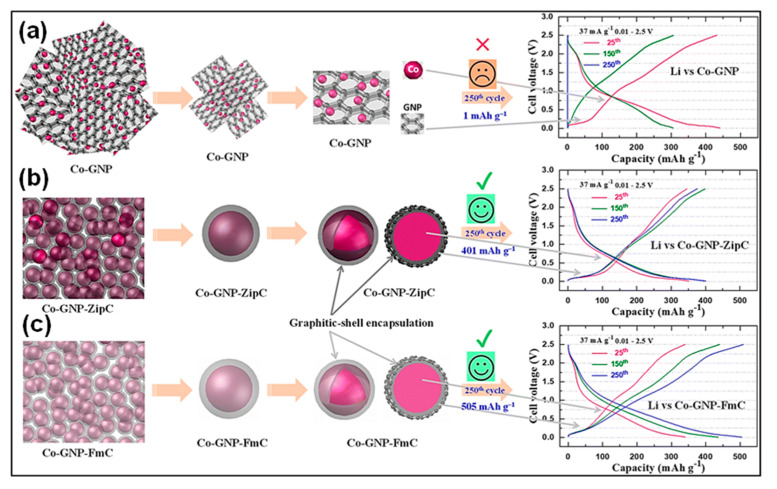
Improved Li+ storage behavior of plastic waste-derived yolk–shell architectures: illustrations of (**a**) Co-GNP (consisting of Co NPs and GNPs) showing reduced Li^+^ storage at the 250th cycle, (**b**) PE-based Co-GNP-ZipC delivering a charge capacity of 377 mAh g^−1^ at the 250th cycle, and (**c**) PS-based Co-GNP-FmC exhibiting a higher capacity of 509 mAh g^−1^ at the same cycle. Adapted from [146]. Copyright 2024, Royal Society of Chemistry.

Most studies on upcycling plastic wastes to hydrogen and CNMs rely on costly techniques that involve multiple processing conditions. The transformation of plastic waste into value-added C is typically neither direct nor simple, often requiring high-temperature reactor treatment or prolonged acid processing. Pol and colleagues [146] reported an ultrafast catalytic MW strategy to convert waste plastics, such as PE Ziploc bags and PS packaging foam, into graphitic shell-entrapped cobalt nanoparticles (Co NPs) within 2 min. When evaluated as LIB anodes, the obtained heterostructures (Co-GNP-ZipC and Co-GNP-FmC) delivered reversible capacities of 377 and 509 mAh g^−1^, respectively. Notably, a CE of 101% was maintained at the 250th cycle, markedly exceeding the performance of bare Co-GNP, which exhibited negligible capacity (<1 mAh g^−1^) (Figure 9a–c). The graphitic core–shell structure effectively protects Co NPs from direct electrolyte exposure, suppresses undesirable side reactions, and improves long-term phase stability and Li^+^ storage performance.

#### 4.1.2. Sodium-Ion Batteries

SIBs have garnered increasing attention in recent times due to the natural abundance of Na resources and their technological similarity to LIBs in terms of fabrication methods [165,166]. The fundamental components and energy storage mechanisms of SIBs closely resemble those of LIBs. Nevertheless, the larger ionic radius of Na^+^ compared to Li^+^ results in distinct ion transport behavior, phase evolution, and solid-electrolyte interphase (SEI) formation. A range of carbonaceous materials, including graphite, PC, and hard C, has been investigated as anode candidates for SIBs [167,168]. Representative studies on waste plastic-derived carbons applied as SIB anodes are summarized in Table 2. Various waste plastics, for instance, polyolefins (PP, PE, PS), ester-containing polymers (PC, PET), and end-of-life tires, have been employed as C sources. High-temperature carbonization remains the primary approach for producing C anode materials for SIB applications.

**Figure 10:**
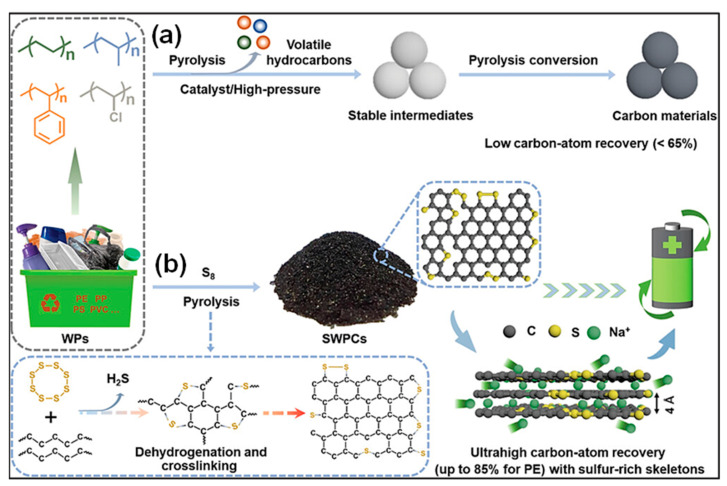
Illustrative comparison of (**a**) traditional annealing strategy employing catalysts and/or high-pressure conditions and (**b**) S-assisted annealing for converting waste plastics into value-added C samples. Adapted from [154]. Copyright 2023, Wiley-VCH.

Deng and co-workers [147] transformed waste PS cups to disordered C by conducting carbonization in a sealed reactor under reasonable temperatures and elevated pressure. When evaluated as an anode material for SIBs, the resulting C exhibited a capacity of 116 mAh g^−1^ over at least 80 cycles. Typically, polyolefin-based plastics such as PE, PP, and PS undergo complete thermal decomposition into volatile HCs during direct annealing, leaving minimal solid C residue. To enhance C yield, catalysts and/or high-pressure systems are often introduced to stabilize intermediate polymeric species and promote C formation (Figure 10a). Nevertheless, the subsequent removal of catalysts and the requirement for stringent annealing conditions increase both environmental burdens and economic costs. Furthermore, these thermolysis-based approaches generally suffer from low C atom utilization efficiency, resulting in significant resource loss and elevated greenhouse gas (GHG) emissions. Wu’s team [154] proposed a S-assisted annealing approach as a straightforward and versatile method for converting waste plastics into value-added C materials with a C-atom recovery as high as 85% (Figure 10b). The resulting sulfur-enriched plastic waste derived carbons (SWPCs) possessed S-doped frameworks with expanded interlayer spacing, which facilitated Na^+^ storage when applied as anodes in SIBs. Remarkably, the initial reversible capacities reached 662 mAh g^−1^ for SWPEC, 578 mAh g^−1^ for SWPPC, and 661 mAh g^−1^ for SWPSC, significantly surpassing those of traditional hard carbons, which typically exhibit capacities of ≤300 mAh g^−1^.

**Figure 11:**
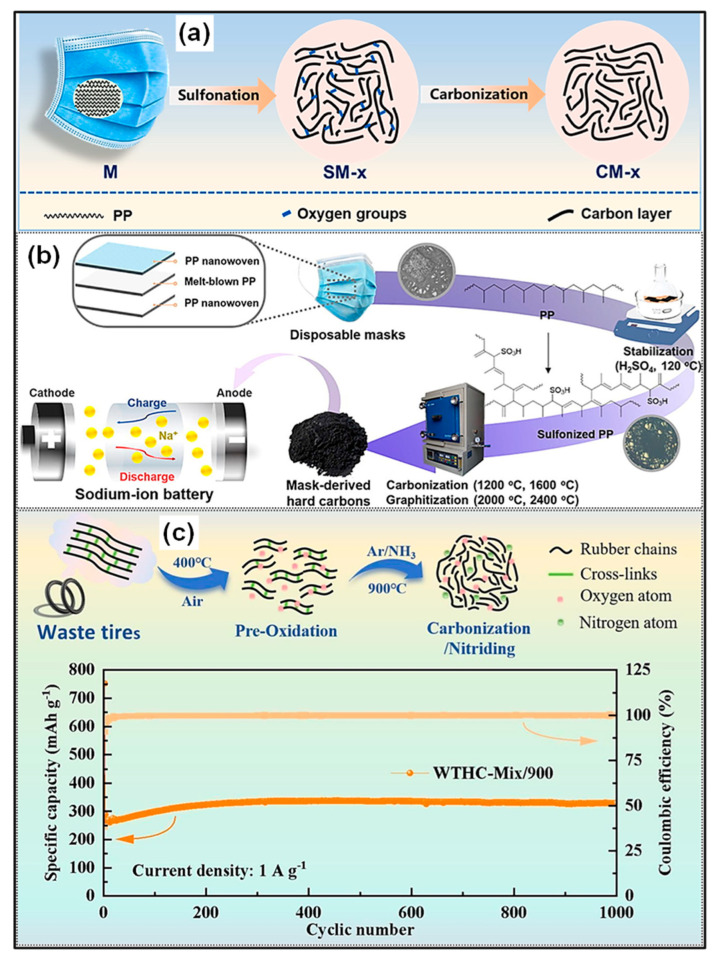
Pictorial illustrations of (**a**) sulfonation-assisted transformation of discarded masks into hard C samples (CM-x). Adapted from [155]. Copyright 2023, Elsevier B.V. (**b**) Preparation of hard C anodes from waste masks via sulfonation treatment. Adapted from [156]. Copyright 2022, Elsevier B.V. (**c**) Fabrication route of nitrogen or oxygen co-enriched hard C derived from waste tires through sequential pre-oxidation and nitridation for SIB anodes. Adapted from [157]. Copyright 2025, Elsevier B.V.

Hard carbons, distinguished by their disordered architectures and expanded interlayer distance, are widely recognized as promising anode candidates for SIBs [127]. They are typically produced via the thermal decomposition of organic precursors, where the selection of feedstock critically influences the resulting nanostructure, microstructural features, pore distribution, and defect density. In contrast to natural polymers, which exhibit fixed monomer compositions and rigid backbone architectures, synthetic polymers provide enhanced structural flexibility through controllable polymerization processes. As a result, synthetic polymers, including plastic materials, have been extensively utilized as precursors for fabricating hard C anodes in SIB systems [169]. Generally, different categories of polymers, including polyolefins (PP, PE, PS) [155,156,157], ester-containing polymers (PC, PET) [158,159,160], and phenolic resins [161,170] require distinct strategies for the preparation of hard carbons. For instance, Xu and co-workers [155] synthesized hard C from waste PP masks through a combined sulfonation and carbonization activity (Figure 11a). The sulfonation step enhanced thermal stability of PP chains, thereby suppressing total decomposition and excessive gas evolution during carbonization. Moreover, the introduction of oxygen-containing functional groups promoted cross-linking among polymer chains, inhibiting graphitic rearrangement and increasing structural disorder. The resulting hard C (CM-180), characterized by a great degree of disorder and limited surface defects, delivered a Na storage capacity of 327.4 mAh g^−1^ along with exceptional cycling stability and rate performance. When assembled into a full cell with an O_3_-NaNi_1/3_Fe_1/3_Mn_1/3_O_2_ cathode, the device achieved an energy density of 238 Wh kg^−1^ and maintained a capacity of 75 mAh g^−1^ even at a high rate of 50 C, demonstrating remarkable rate capability. Lee and colleagues [156] similarly prepared hard C from discarded PP masks through H_2_SO_4_ treatment followed by high-temperature annealing up to 2400 °C (Figure 11b). Extending the sulfonation duration significantly increased the C yield, reaching as high as 50%, suggesting that the formation of thermally stable, infusible structures during sulfonation was crucial for C framework development. The acid treatment not only promoted cross-linking reactions but also facilitated the generation of PAH species, which were reflected by the pronounced D and G bands in the Raman plot. Notably, even heat treatment at 2400 °C failed to induce graphitic ordering, indicating the inherently non-graphitizable nature of PP-derived C. Electrochemically, the hard C anode delivered a reversible capacity of 340 mAh g^−1^ at 0.01 A g^−1^ and retained 53% of this capacity when the current density was increased by two orders of magnitude, demonstrating excellent rate performance. A full cell assembled with this anode achieved a reversible capacity of 110 mAh g^−1^ and an energy density of 352 Wh kg^−1^, confirming its potential for SIB applications. Moreover, further enhancement of hard C characteristics can be realized through optimization of preparation parameters. Huang’s team [157] synthesized nitrogen- and oxygen-enriched hard C from waste tires through a two-stage process involving pre-oxidation followed by nitridation (Figure 11c). The electrochemical performance of the resulting material was improved through multiple synergistic approaches, for instance, pre-oxidation to generate additional active channels, precise regulation of annealing temperature and duration to tailor the microstructure and pore distribution, and nitrogen doping to further enhance the density of electrochemically active channels. When evaluated as an anode for SIBs, the material delivered a stable discharge capacity of 406.7 mAh g^−1^ over 100 cycles at a current density of 1 A g^−1^.

**Figure 12:**
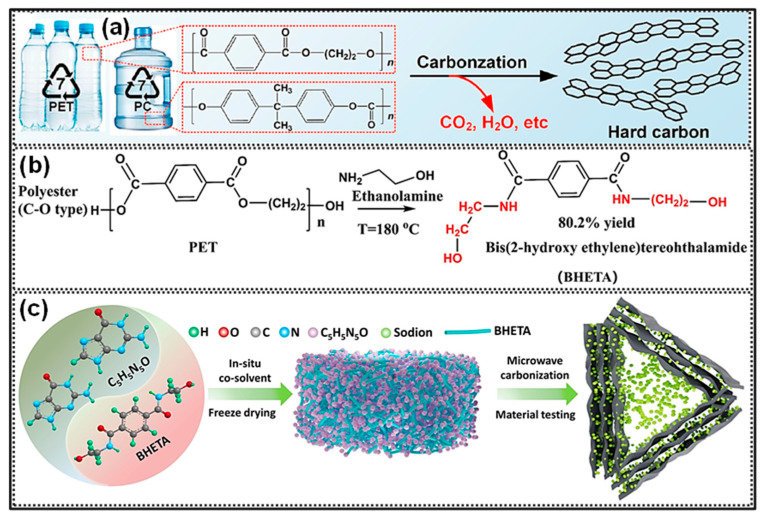
(**a**) Diagram showing the conversion of ester bond-containing plastics (PC, PET) into hard C via direct carbonization for SIB applications. Adapted from [158]. Copyright 2021, Elsevier B.V. (**b**) Reaction mechanism illustrating PET depolymerization to BHETA, and (**c**) schematic of the subsequent hard C preparation route. Adapted from [160]. Copyright 2024, Elsevier B.V.

The selection of appropriate precursors is crucial for the preparation of high-performance hard C anodes. The chemical composition of the starting materials strongly affects the resulting local microstructure, which in turn governs Na+ storage behavior. In particular, oxygen-containing species in the precursor can inhibit graphitic rearrangement during pyrolysis, promoting the formation of greatly turbostratic C structures. Consequently, plastics rich in ester linkages, for instance, PC and PET, are considered promising feedstocks for synthesizing advanced hard C anodes [158]. Guo and co-workers [158] synthesized hard carbons from waste PC and PET through direct annealing at 1400 °C (Figure 12a). The resulting PC- and PET-derived hard C anodes delivered capacities of 327 mAh g^−1^ and 342 mAh g^−1^ at a current density of 20 mA g^−1^, with ICE of 84.7% and 86.1%, respectively. Notably, both materials exhibited stable cycling behavior, showing negligible capacity fading over 140 cycles at 0.1 A g^−1^. It is important to note that the Na storage behavior of hard C strongly depends on its microstructural characteristics, which are governed by precursor chemistry and carbonization parameters. Elevated pyrolysis temperatures are generally necessary to generate numerous closed pores, a critical feature for achieving high-performance hard C anodes in SIBs [171].

Yang’s team [159] investigated the transformation of PET into hard C through both traditional thermal treatment and microwave (MW) carbonization at temperatures below 1000 °C. During traditional thermal decomposition, PET undergoes pyrolysis, polycondensation, and structural rearrangement of its benzene rings and hydroxyl-containing units, ultimately forming hard C. In contrast, MW carbonization benefits from the strong MW absorption capability of PET due to its abundant hydroxyl groups. Dielectric loss under MW irradiation enhances reaction kinetics and stimulates the development of abundant pore structures. Multiple reflections and refractions of MWs within these open pores generate localized “hot spots”, thereby accelerating the transformation of PET to hard C with a dense cellular framework. As a result, two distinct hard C structures were obtained: one featuring pseudo-graphitic domains and the other enriched with closed pores. By simply adjusting the carbonization approach at relatively low temperatures, hard carbons with different microstructures can be produced. Notably, the MW-900 sample, characterized by a higher proportion of closed pores, delivered a capacity of 344 mAh g^−1^. The study further demonstrated that Na storage in the low-voltage plateau region originates from interlayer intercalation and closed-pore filling mechanisms [159].

Beyond structural engineering, diverse C precursors and post-treatment strategies have been explored to fabricate hard carbons with varying degrees of heteroatom incorporation. Such modifications can accelerate Na+ diffusion kinetics, thereby improving capacity and rate capability [172]. However, straightforward physical mixing approaches, including template-assisted synthesis and ball milling, often result in substantial heteroatom loss during high-temperature carbonization. In addition, post-synthesis doping may produce non-uniform heteroatom distribution, which can compromise structural integrity and increase the likelihood of framework collapse [173]. In a recent study, Guo’s team [160] reported an in situ strategy for producing greatly nitrogen-doped, interconnected honeycomb-like hard carbons from PET. In this method, PET first underwent aminolysis to generate nitrogen-containing intermediates (BHETA), which were integrated with guanine (Gua) acting as a 2D self-template (Figure 12b). Subsequently, nitrogen-doped hard C was obtained through co-dissolution and freeze-drying of BHETA and Gua, followed by MW-assisted carbonization (Figure 12c). Incorporation of pyrrolic N_5_ and pyridinic N_6_ effectively enlarges the interlayer distance during Na^+^ insertion and extraction, thereby improving electrochemical reactivity. The resultant hard C demonstrated an increase in the Na^+^ diffusion coefficient by approximately 1.5 orders of magnitude (10–8.2 vs. 10–9.76 cm^2^ s^−1^). As a result, it delivered a reversible capacity of 452 mAh g^−1^ at 0.02 A g^−1^ and maintained 388 mAh g^−1^ at 0.5 A g^−1^, indicating excellent rate capability. Furthermore, the electrode retained 87.6% of its capacity after 2000 cycles at 0.5 A g^−1^. The assembled full cell achieved 91.8% capacity retention after 200 cycles at 0.1 A g^−1^, while the pouch cell configuration preserved 90.7% after 100 cycles at 0.2 A g^−1^.

**Figure 13:**
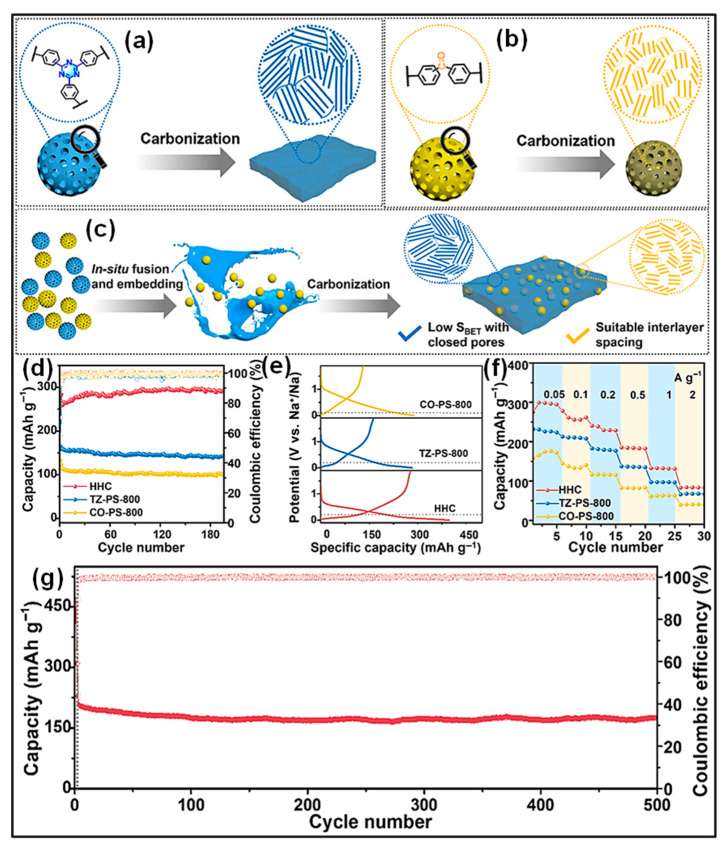
Schematic illustration of preparation mechanisms for (**a**) TZ-PS-derived C, (**b**) CO-PS-derived C, and (**c**) HHC. Electrochemical performance comparisons, including (**d**) cycle stability and (**e**) first-cycle galvanostatic charge–discharge profiles at 0.02 A g^−1^, (**f**) rate capability from 0.05 to 2 A g^−1^, and (**g**) long-term cycling behavior of HHC at 1 A g^−1^. Adapted from [174]. Copyright 2024, Wiley-VCH.

The realization of economically viable and high-performance hard C anodes is critical for the further advancement of SIB systems. Nevertheless, simultaneously attaining high ICE and substantial sodium storage capacity through low-temperature annealing remains challenging, mainly due to the presence of excessive structural defects and insufficient formation of closed pores. Wang and colleagues [174] prepared a hybrid hard C (HHC) derived from PS containing specific molecular bridge structures through an in situ fusion and embedding strategy at a relatively low annealing temperature of 800 °C. The obtained HHC featured low SSA, numerous closed pores, and an embedded spherical morphology. During the carbonization process, triazine-crosslinked PS (TZ-PS) spheres underwent in situ fusion, forming a stable layered framework characterized by reduced SSA and a high density of closed pores (Figure 13a). In contrast, carbonyl-crosslinked PS (CO-PS) spheres retained their spherical morphology, displaying higher SSA and adequate interlayer distance (Figure 13b). As a result, co-carbonization of hypercrosslinked PS generates a HHC characterized by low SSA, abundant closed pores, and defect-derived nanopores (Figure 13c). This differs from conventional hybrid carbons, which are typically obtained by carbonizing separate precursors that possess large interfacial barriers and sizeable domain structures. Conversely, in situ fusion and embedding of PS templates promote close interfacial integration and strengthened structural cohesion within the HHC framework. Benefiting from this architectural feature, the HHC exhibited enhanced Na^+^ storage behavior, delivering higher ICE of 70.2% and a greater capacity of 279.3 mAh g^−1^ compared with CO-PS-800 (132.1 mAh g^−1^) and TZ-PS-800 (165 mAh g^−1^) (Figure 13d–f). Additionally, it maintained 85% capacity retention at 1 A g^−1^ over 500 cycles (Figure 13g).

#### 4.1.3. Potassium-Ion Batteries

PIBs are regarded as attractive candidates for large-scale energy storage owing to the abundance of K-resources, their low standard redox potential (−2.94 V vs. SHE), and electrochemical characteristics comparable to Li [130]. Nevertheless, the relatively large ionic radius of K^+^ (1.38 Å) induces significant structural variation in electrode materials and slow reaction kinetics during insertion and extraction, which negatively affects battery behavior [125]. Consequently, the development of high-performance electrodes for PIBs is urgently required. The favorable intercalation of K into low-cost, scalable graphite-like carbons has further stimulated interest in PIB systems [175]. Among potential anodes, C-based materials stand out owing to their economic viability, natural abundance, good ionic conductivity, and robust physicochemical stability [176]. To date, however, only limited studies have explored the conversion of waste plastics into C products for PIB anodes [157,177,178,179]. Qian’s team [178] synthesized a sandwich-like architecture composed of porous CNSs supporting hexagonal C flakes using PE as the C precursor and magnesium as the inducing agent via a single-stage hydrothermal treatment at 700 °C. Structural analysis revealed that hexagonal C flakes possessed a preferred (002) orientation, exposing numerous edge-active channels and shortening the diffusion pathway for K^+^ ions. Additionally, the interconnected porous CNSs facilitated rapid ion transport and enhanced the capacitive contribution. As a result, the porous CNSs anode delivered a reversible capacity of 528.7 mAh g^−1^ at 0.2 A g^−1^, maintained 152.7 mAh g^−1^ at 10 A g^−1^, and exhibited remarkable long-term stability with 112.1 mAh g^−1^ retained at 5 A g^−1^ after 10,000 cycles.

Hard carbons have emerged as attractive anode candidates for PIBs [157,180]. Liu’s group [180] prepared a S-rich hard C using PS and elemental S as precursors. When evaluated as a PIB anode, the S-doped hard C exhibited outstanding cycling durability and rate capability, delivering 298.1 mAh g^−1^ at 0.1 A g^−1^ over 1000 cycles with a capacity retention of 95.2%, and maintaining 220.2 mAh g^−1^ at 0.5 A g^−1^ after 5200 cycles. The electrode demonstrated remarkable stability under low and high current densities. Structural and kinetic analyses indicated that enlarged interlayer distance (0.382 nm) facilitated rapid K^+^ diffusion and mitigated volume expansion, thereby preserving structural integrity during potassiation and depotassiation. Furthermore, DFT measurements revealed that S dopants introduce abundant active channels for K^+^ adsorption, contributing to enhanced reversible capacity.

Sreeraj and co-workers [181] prepared hard C through the direct carbonization of PVC. When evaluated as PIB anodes, both commercial and waste-derived PVC carbons delivered reversible capacities of 477 and 378 mAh g^−1^, respectively, at 0.1 C. In addition, waste PET was transformed into hard C via a one-step carbonization route. The direct pyrolysis of PET enlarged the interlayer distance and generated partially closed slit-like micro- and meso-pores, which markedly enhanced the low-voltage plateau capacity, contributing 68% of total capacity. The PET-derived hard C carbonized at 800 °C achieved a capacity of 305 mAh g^−1^, with 32% of the capacity originating from the battery-like low-voltage plateau [182].

Huang’s team [157] prepared nitrogen/oxygen co-enriched hard C from waste tires through a two-stage strategy involving pre-oxidation followed by nitridation. Improved electrochemical properties were attributed to synergistic effects: pre-oxidation introduced additional active channels, precise control of pyrolysis temperature and duration optimized the microstructure and pore distribution, and nitrogen doping further increased electrochemically active channels. When applied as a PIB anode, the material delivered a reversible capacity of 363 mAh g^−1^ after 200 cycles at 0.1 A g^−1^ and retained 328.9 mAh g^−1^ after 1000 cycles at 1 A g^−1^, demonstrating excellent cycling durability. In addition to hard C, other waste plastic-derived carbons, including soft C [179] and graphitic C [177], have been explored as PIB anodes. Compared with hard C, soft C contains abundant short-range ordered graphite-like domains, which facilitate K^+^ transport and improve intercalation/deintercalation kinetics, thereby enhancing rate performance [179]. Notably, PVC templates can be carbonized at different temperatures to tailor defect density and crystalline structure in soft carbons.

Zhang’s group [179] systematically examined how carbonization temperature influences the crystalline characteristics of soft C materials. When treated at 800 °C, the resulting soft C exhibited a highly defective structure with short-range ordering, providing abundant adsorption and intercalation channels for K^+^ ions and delivering a capacity of 302 mAh g^−1^. In contrast, carbonization at 1200 °C produced a material with enhanced crystallinity and a greater proportion of short-range ordered graphene domains, leading to an extreme intercalation capacity of 208 mAh g^−1^.

### 4.2. Cathodes for Lithium-Ion Batteries

**Figure 14:**
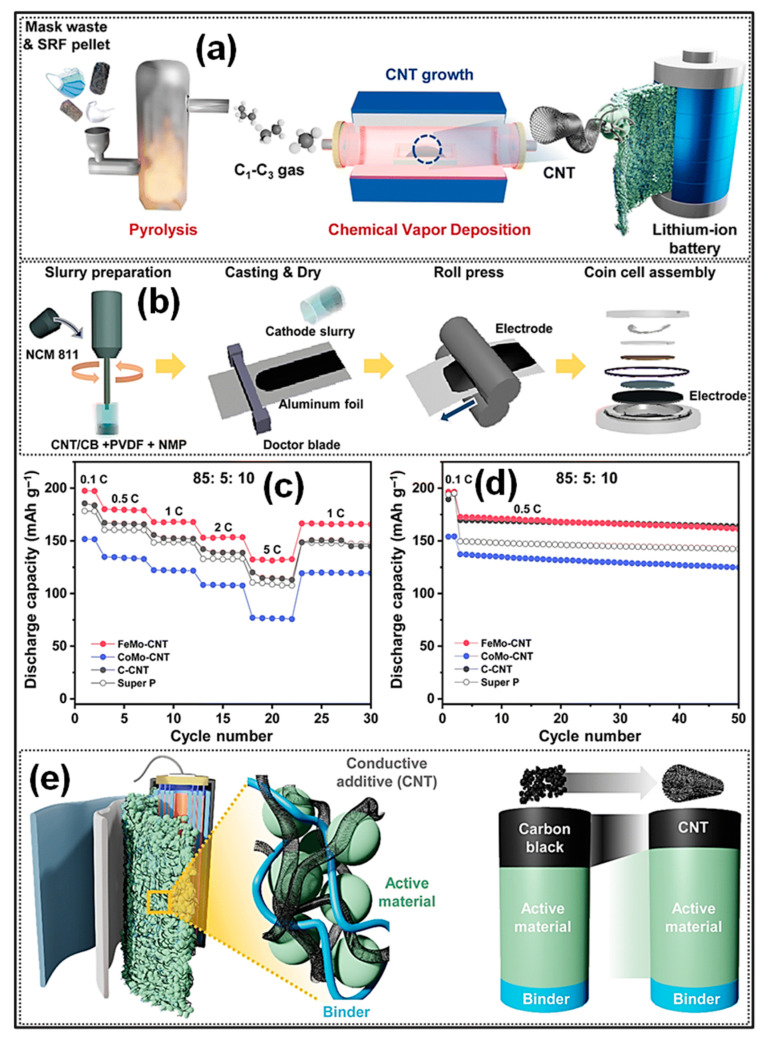
Pictorial representations of (**a**) conversion of plastic waste into CNTs through a combined annealing-CVD route for enhancing LIB behavior, (**b**) incorporation of CNTs as conductive additives in LIB cathodes, (**c**) rate capability and (**d**) cycle stability of NCM811-based cathodes with a composition of active material/C additive/binder = 85:5:10 (wt %), and **(e**) structural configuration of a LIB, highlighting the distribution of conductive additives within the electrode. Adapted from [183]. Copyright 2023, Royal Society of Chemistry.

CNTs have been widely recognized as effective conductive additives for cathode materials in LIBs. By establishing interconnected conductive pathways among active particles, such additives enhance the overall electronic conductivity of the electrode. Due to their large aspect ratio, extensive SSA, and low intrinsic electrical resistance, CNTs are regarded as superior alternatives to conventional C black (CB). Substituting CB with plastic-derived CNTs can potentially increase the proportion of electrochemically active material in the electrode, thereby improving the overall characteristics of LIBs. An’s group [183] synthesized CNTs from discarded PP masks and solid recovered fuel (SRF, composed of PP, PE, PS, and PET) through a combined annealing-CVD approach. The C_1_-C_3_ HCs generated during annealing served as effective C precursors for CNT growth via CVD, enabling their application as conductive additives in LIB cathodes (Figure 14a). As-prepared CNTs were mixed with the active material LiNi_0.8_Co_0.1_Mn_0.1_O_2_ (NCM811) and dispersed in a PVDF binder solution (Figure 14b). The resulting slurry was coated onto aluminum foil to form the cathode, and coin cells were assembled using Li metal as the counter electrode. Electrochemical evaluation under various C-rates and cycling conditions demonstrated that mask-derived CNTs (FeMo-CNTs) outperformed conventional C-CNTs and Super P when an appropriate mixing ratio was employed (Figure 14c,d). To enhance active material loading and overall cell performance while minimizing the content of conductive C (Figure 14e), careful optimization of cathode composition and homogeneous component distribution is necessary. Future efforts should focus on increasing the added value of waste plastic-derived CNTs as high-efficiency conductive additives for LIBs.

### 4.3. Cathodes for Li-S Batteries

Rechargeable Li-S batteries (LSBs) are considered strong candidates for next-generation energy storage systems due to their large theoretical specific capacity (1675 mAh g^−1^, depending on S), elevated energy density (2600 Wh kg^−1^), natural availability, and economic viability of S [184]. Nevertheless, their practical implementation has been limited by the absence of suitable electrode materials for both the cathode and anode. Significant advancements have been made in enhancing LSB performance through electrode engineering, particularly via the integration of functional C products. These C-based materials typically function as conductive frameworks for S cathodes or as interlayers and coatings on separators [132]. Representative examples, for instance, graphene, CNTs, CSs, PCs, and other structurally diverse C architectures [185]. Porous carbons, particularly HPCs, have gained increasing attention due to their high SSA, large pore volume, low density, chemical stability, and well-defined multimodal pore structures [132]. Compared with C materials possessing a single pore size distribution, HPCs exhibit distinct advantages in LSB systems. When employed as S hosts, their conductive C framework enhances the overall electrical conductivity of the cathode. In addition, the presence of micro- and meso-pores increases the accessible SSA and provides abundant active sites for S redox reactions. Meanwhile, the large meso- and macro-pores contribute substantial pore volume, enabling high S loading and accommodating the volumetric expansion during cycling [132].

Studies on waste plastic-derived C products applied in LSBs are summarized in Table 4. The development of HPCs with heteroatom incorporation, capable of providing both physical confinement and chemical anchoring of polysulfides at reasonable cost, is essential for promoting the practical deployment of LSBs. Pol and co-workers [186] reported the fabrication of porous sulfonated C from waste LDPE through MW-assisted sulfonation. MW irradiation accelerated the sulfonation of LDPE and simultaneously generated abundant pore structures. When employed as an interlayer in LSBs, the material enabled the S cathode to deliver a capacity of 776 mAh g^−1^ at 0.5 C, with 79% capacity retention after 200 cycles.

Sun’s team [187] prepared hierarchically N,S-codoped carbons from PVC via KOH-assisted carbonization in the presence of urea. The resulting N,S-codoped HPCs were utilized as S host materials and exhibited reversible capacities of 1205 mAh g^−1^ at 0.1 C and 836 mAh g^−1^ at 1 C. Notably, a capacity of 550 mAh g^−1^ was retained after 500 cycles at 1 C. The outstanding cycle stability was credited to the combined effect of hierarchical pore architecture and dual heteroatom doping, which facilitated both physical confinement and chemical anchoring of intermediate lithium polysulfides (LiPSs). Chu’s group [188] synthesized S,P-codoped C from waste PS foam through a sulfonation-assisted annealing process. When applied in LSBs, the highly porous framework enabled substantial S loading and effective physical confinement of LiPSs, while dual S and P doping improved electronic conductivity and strengthened chemical anchoring of polysulfide intermediates. The S@S,P-codoped porous C cathode with a S loading of 2 mg cm^−2^ delivered an initial capacity of 893 mAh g^−1^ at 2 C, with a low-capacity fading rate of 0.049% per cycle over 800 cycles. At a higher S loading of 4.8 mg cm^−2^, the cathode maintained excellent rate capability and cycling stability, showing a decay rate of 0.06% per cycle over 600 cycles at 2 C. Furthermore, under lean electrolyte conditions (E/S ratio of 5 mL g^−1^), a capacity of 694 mAh g^−1^ was retained after 150 cycles at 0.5 C.

**Figure 15:**
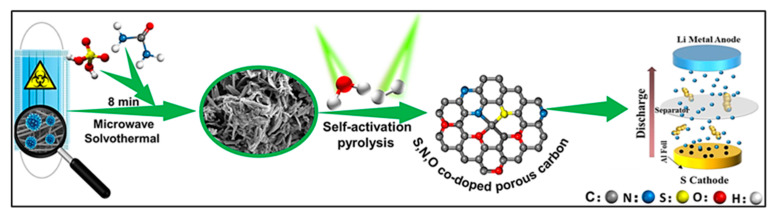
Illustration of the fabrication process of S/N/O-codoped C derived from waste masks through MW-assisted solvothermal process and subsequent self-activation annealing for LSB applications. Adapted from [193]. Copyright 2022, Elsevier B.V.

Additionally, Elumalai and colleagues [189] prepared N,S-codoped C derived from waste PS for application in LSBs. The electrode exhibited a stable discharge capacity of 1079 mAh g^−1^ at 0.1 C and maintained long-term cycling behavior over 500 cycles. The enhanced stability was attributed to the mesoporous C framework containing thiophene functionalities, which effectively suppressed LiPSs dissolution through strong chemical interactions with Li_2_S_n_ species. Furthermore, Guo’s team [192] transformed discarded PP face masks into HPCs for LSBs. MW-driven treatment with concentrated H_2_SO_4_ and urea enabled simultaneous sulfonation, oxidation, and nitridation of PP, thereby enhancing its thermal stability and introducing S, N, and O heteroatoms. Subsequent self-activation yielded porous C materials (Figure 15). Benefiting from the synergistic effects of multi-heteroatom incorporation and hierarchical porosity, the C obtained at 900 °C delivered an initial discharge capacity of 1459.8 mAh g^−1^ at 0.1 C and retained 52.3% of its capacity after 400 cycles at 0.5 C.

### 4.4. Cathodes for Zn-Air Batteries (ZABs)

Rechargeable ZABs have attracted significant attention as prospective next-generation energy storage technologies, owing to their large theoretical energy density, inherent safety, and structural flexibility [87,194]. The overall performance of ZABs is primarily dictated by the ORR and OER that occur at the air cathode. In these systems, integration of bifunctional electrocatalysts within the air cathode is essential for ensuring prolonged cycling stability and high energy efficiency [195]. Among various catalyst materials, C-based electrocatalysts have attracted extensive interest owing to their low cost, high electrical conductivity, large SSA, tunable electronic properties, and structural diversity [196]. Recently, significant efforts have focused on deriving C-based catalysts from waste plastics, including porous carbons [197,198] and CNTs [199,200,201] based materials.

Innocenti’s group [202] transformed waste tire-derived char into active ORR electrocatalysts applicable to alkaline fuel cells and ZABs. The char produced via MW-assisted annealing exhibited an ORR onset potential of −90 mV versus RHE and followed a favorable four-electron transfer pathway. The enhanced catalytic performance was ascribed to its high SSA and the presence of ZnO NPs uniformly dispersed within the C framework. Similarly, Lee and co-workers [203] prepared nitrogen-doped C catalysts from rubber and CB components of waste tires through sulfonation treatment followed by carbonization under an NH_3_/N_2_ environment. The introduction of nitrogen functionalities significantly improved ORR behavior compared with non-sulfonated carbonized tire samples.

Durante and co-workers [204] prepared Fe-N-C catalysts from both PE and PU wastes by introducing FeCl_3_ to facilitate the formation of Fe-N*_x_* active centers. The ORR catalytic behavior and selectivity were found to depend strongly on the nature of the active species, including Fe-N*_x_* moieties, Fe_3_C, Fe@C, pyrrolic-N, and pyridinic-N sites. These sites contribute differently to ORR catalysis and may undergo activation or deactivation depending on the electrolyte pH. In a related study, Zhang’s team [205] constructed isolated Fe-N*_x_* channels in conjunction with Fe_3_C NPs co-integrated within N-doped C derived from waste PET. Benefiting from synergistic interaction among Fe-N*_x_* centers and Fe_3_C species, along with the hierarchical pore architecture, the catalyst demonstrated superior ORR behavior. Overall, the ORR behavior of porous C-based electrocatalysts can be enhanced through heteroatom incorporation (i.e., N, S) [198] and the incorporation of transition-metal active channels such as Fe [205].

**Figure 16:**
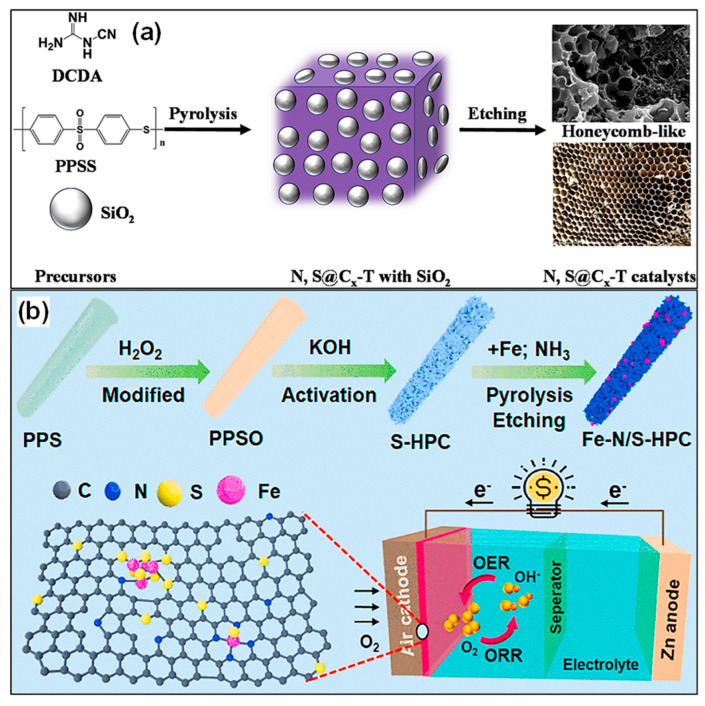
Pictorial illustration of the fabrication routes for (**a**) PPSS-derived N,S co-doped HPC as a metal-free ORR catalyst. Adapted from [207]. Copyright 2020, Elsevier B.V. (**b**) PPS-derived Fe,N,S co-doped C employed as a bifunctional catalyst for ZABs. Adapted from [206]. Copyright 2022, Elsevier B.V.

Heteroatom-doped C electrocatalysts, particularly nitrogen-doped systems, can be synthesized via self-doping strategies employing heteroatom-encompassing plastic waste as C templates [197,206]. Niu and colleagues [207] prepared N,S-codoped C derived from poly (phenylene sulfide sulfone) (PPSS) as a metal-free ORR catalyst. In this process, sulfur atoms inherent in the PPSS backbone served as the sulfur precursor, while dicyandiamide (DCDA) provided nitrogen, and SiO_2_ NPs were employed as a hard template. Pyrolysis under an argon environment followed by template removal yielded N,S-codoped C (denoted as N,S@C) (Figure 16a). By optimizing the SiO_2_ content, the sample carbonized at 1000 °C (N,S@C_M_-1000) exhibited the most favorable ORR activity in alkaline media. When applied as a cathode catalyst in ZABs, it delivered a power density of 90 mW cm^−2^ along with exceptional durability and rate capability.

Li’s group [206] prepared Fe, N, and S co-doped HPC (Fe-N/S-HPC) through a synergistic strategy involving KOH activation and annealing of polyphenylene sulfide (PPS) fibers (Figure 16b). The resulting material possessed an ultrahigh SSA (2223.31 m^2^ g^−1^), which enhanced the electrode-electrolyte interfacial contact. Simultaneously, the incorporation of Fe, N, and S heteroatoms generated abundant catalytically active sites, contributing to the superior catalytic performance. Interestingly, the introduced metal species formed a distinctive core–shell configuration, composed of an Fe_x_O_y_S_z_ shell surrounding an Fe_x_N_y_S_z_ core, which effectively improved both catalytic behavior and structural stability. In alkaline media, Fe-N/S-HPC catalyst demonstrated outstanding bifunctional oxygen electrocatalytic activity, highlighting its potential for ZAB applications.

Additionally, CNTs possess a distinctive tubular architecture, high thermal resistance, and outstanding conductivity, which has stimulated extensive research into converting waste plastics into CNT-based ORR electrocatalysts [199,200,201]. CVD, typically employing HC gases generated from plastic pyrolysis, is the most widely adopted synthesis route. In most cases, CNTs loaded with transition metals like Ni, Co, or Fe exhibit ORR behavior comparable to that of commercial Pt/C catalysts [199,200,201]. The CNT framework functions as an efficient electron-conducting network, facilitating rapid charge transfer and mitigating metal NPs agglomeration. However, despite their promising catalytic activity, the fabrication procedures are relatively complex and prolonged and may involve the emission of hazardous volatile organic products. Likewise, Gao’s group [200] proposed a MW-assisted thermal shock approach to effectively immobilize Fe NPs onto CNT frameworks. The resulting Fe-doped CNTs demonstrated pronounced activity under MW-induced annealing conditions and exhibited a low I*_D_*/I*_G_* ratio of 0.31, indicating a high degree of graphitization. During the pyrolysis-recycling process, the CNTs underwent reconstruction to form an interconnected cross-linked framework, which enhanced electron transport and provided a favorable architecture for electrocatalyst fabrication. Subsequent nitrogen incorporation further enhanced the electrocatalytic properties, achieving an onset potential of 0.923 V versus RHE, thereby demonstrating strong potential for ORR applications.

### 4.5. Prospects for Future Development

**Figure 17:**
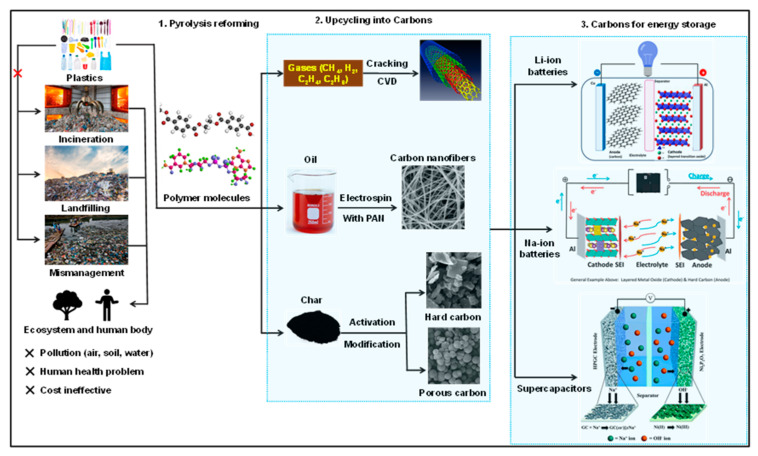
Diagram illustrating the conversion of pyrolysis-derived products from plastic wastes to value-added C products for energy storage devices.

The conversion of plastic wastes to CNMs for rechargeable battery anodes represents a frontier approach that bridges resource recovery with advanced energy storage technologies. Future investigations should prioritize performance enhancement, environmentally benign synthesis strategies, and pathways toward scalable industrial deployment. The main research perspectives are summarized as follows. To achieve precise structural regulation, it is essential to design C frameworks with hierarchical micro-/meso-porous architecture that promote rapid ion diffusion. In particular, the targeted objective is to maintain a capacity exceeding 300 mAh g^−1^ for SIB anodes at the current rate of 1 C. Further investigation into N/S/P doping strategies is required to optimize the electrochemical performance of C materials. Tailoring the electronic configuration through heteroatom incorporation can significantly enhance redox activity and improve CE. In addition, interface engineering should be employed to construct C hosts enriched with polar functional groups (e.g., -COOH and -SO_3_H) for LSBs, thereby strengthening LiPS adsorption with binding energies in the range of 1.5–2 eV. To advance sustainable synthesis routes, the development of molten salt-assisted MW carbonization at relatively low temperatures (<600 °C) is highly desirable, as it may reduce energy consumption by more than 50% compared with conventional thermal treatments. Moreover, comprehensive utilization of multiple products generated from waste plastic annealing is recommended to fabricate high-value C products for energy applications (Figure 17). Plastic wastes, consisting predominantly of C and hydrogen, can function as efficient reducing agents for the recycling of spent LIBs via integrated annealing strategies [208]. Under sealed high-pressure conditions, the co-pyrolysis of spent LIBs with LDPE promotes rapid reactions between decomposition-derived gaseous species and Li transition metal oxides. This process enables highly efficient Li recovery (>98%), induces the conversion of transition metals, and catalytically transforms volatile intermediates into solid C products. Remarkably, CNTs were formed in situ on NiCo alloy phases originating from NCM cathode materials, displaying pronounced electrocatalytic activity toward the ORR [208]. Advancing this field requires the integration of materials engineering, green chemistry principles, and intelligent fabrication strategies. Through interdisciplinary collaboration, plastic waste can be upgraded into sustainable C resources for next-generation energy storage applications.

To assess the practical viability of plastic-derived C materials for rechargeable batteries, it is essential to compare their electrochemical performance with that of conventional commercial electrodes. For LIBs, graphite remains the dominant commercial anode material, with a theoretical capacity of 372 mAh g^−1^ and excellent cycling stability [209]. In comparison, plastic-derived carbons often exhibit comparable or higher reversible capacities, particularly when engineered with defect-rich structures and heteroatom doping, which provide additional Li storage sites beyond intercalation mechanisms. However, these materials frequently suffer from lower ICE and higher irreversible capacity loss due to extensive surface reactions and SEI formation. For SIBs, hard C is widely regarded as the most promising commercial anode, typically delivering capacities in the range of ~250–350 mAh g^−1^ with good cycling stability [210]. Plastic-derived carbons, especially those with hierarchical porosity and enlarged interlayer spacing, have demonstrated competitive Na-storage performance, in some cases approaching or exceeding that of conventional hard C. Nevertheless, challenges such as low initial efficiency, structural instability, and scalability remain. Overall, while plastic-derived C materials show significant promise due to their tunable microstructure and sustainable origin, further optimization is required to achieve performance parity with established commercial materials, particularly in terms of long-term stability, ICE, and large-scale reproducibility.

## 5. Summary and Future Outlook

The conversion of waste plastics into hydrogen and C-based materials has emerged as a promising approach for achieving both resource recovery and the production of high-value materials. Owing to the adjustable physicochemical properties of plastic-derived carbons, considerable progress has been made in their application to electrochemical energy storage. This review provides a comprehensive overview of thermochemical strategies, including catalytic decomposition and carbonization, for transforming waste plastics into functional C materials. Particular attention is given to recent developments in producing diverse C architectures, such as graphene, CNTs, CSs, CNSs, PCs, and their hybrid structures. Furthermore, advances and ongoing challenges associated with their utilization in SCs and rechargeable battery systems are critically discussed. The main conclusions are summarized as follows:

(1) To establish sustainable pathways for converting waste plastics into high-performance C electrodes, careful consideration must be given to structural evolution during processing. Variations in geometrical morphology can lead to distinct intrinsic C properties, including differences in electron transport pathways, surface-to-volume ratios, and distribution of atomic-scale active centers. Consequently, the deliberate design of tailored C architecture must be closely matched to the requirements of specific applications. Achieving targeted C products requires precise regulation of key parameters that govern the final composition and structural characteristics. Moreover, a deeper understanding of the transformation mechanisms from plastics to C under various reaction conditions is crucial.
(2) Fabrication of CNMs with diverse dimensional architectures from plastic waste typically involves complex procedures and specialized equipment. Developing highly selective catalysts that enable elevated C yields during carbonization remains a significant challenge. In addition, effective utilization of valuable byproducts (i.e., hydrogen) and proper management of harmful emissions (e.g., toxic volatile compounds) must be carefully addressed. For energy storage applications, C materials are generally required to meet stricter criteria, including thorough purification and structural uniformity. Therefore, comprehensive techno-economic evaluations and life-cycle assessments covering the entire process, from waste plastic treatment to C electrode manufacturing, should be conducted in upcoming studies.
(3) CNMs derived from plastic wastes, particularly heteroatom-incorporated HPCs, have demonstrated remarkable electrochemical characteristics as SC electrodes. Through thermochemical routes such as pyrolysis, activation, and heteroatom incorporation, polymers including PET and PE can be transformed to HPCs with SSAs exceeding 1500 m^2^ g^−1^. When integrated with TM hydroxides, for example, Ni-Co-layered double hydroxides, these composites exhibit enhanced specific capacitance, elevated energy density, and robust cycling durability. Moving forward, research efforts should focus on environmentally sustainable approaches, such as low-temperature catalytic cracking and electrochemical reforming, to reduce both energy consumption and production costs. Additionally, optimizing pore architectures (i.e., hierarchical micro-/meso-porous structures) and tailoring surface functionalities (e.g., N and O incorporation) are essential to improve charge transport and interfacial adhesion. Establishing an integrated industrial framework encompassing plastic recycling, C material synthesis, and device assembly will also be crucial to address challenges related to mechanical robustness and batch consistency.
(4) Significant advances have been achieved in converting waste plastics into carbonaceous materials for alkaline-ion battery anodes, including LIBs and SIBs. Technologies such as catalytic annealing and flash carbothermal reduction enable the transformation of plastic waste into hard carbons and porous carbons suitable for anode applications. In particular, CNTs synthesized via CVD using pyrolysis gases from waste plastics exhibit strong potential as LIB anode materials. For SIB applications, waste plastic-derived hard carbons can deliver high reversible capacities and excellent cycling stability comparable to commercial counterparts. The selection of appropriate polymer precursors is critical for fabricating high-performance hard C anodes, as the precursor chemistry strongly influences the resulting microstructure and Na storage behavior. Notably, oxygen-containing precursors can inhibit graphitic rearrangement during pyrolysis, promoting the formation of turbostratic structures favorable for Na^+^ storage. Compared with polyolefins, polyester-based plastics (i.e., PC and PET) demonstrate greater promise for producing high-performance hard C.
(5) Primary routes for converting plastic waste into CNMs encompass pyrolysis, molten salt-mediated catalysis, chemical activation, and advanced techniques such as FJH- and MW-assisted processing. Among these, pyrolysis remains the most industrially mature and widely implemented technique, with multiple projects exceeding capacities of 10,000 tons per year operating globally and demonstrating strong feedstock adaptability. Nevertheless, flue gas purification units contribute roughly 25% of the overall capital expenditure [211,212]. Economically, integrating C production with olefin co-generation can lower costs to approximately 590 Euro per ton, whereas a dedicated C production pathway results in an estimated cost of 980 Euro per ton. The molten salt approach shows significant potential for further cost reduction, although its salt recovery systems are currently limited to single-line production capacity. When the recycling efficiency of salts including KCl or K_2_CO_3_ reaches approximately 95%, PC with SSA of 1800 m^2^ g^−1^ can be manufactured at an estimated cost of 1350 Euro per ton. Chemical activation remains highly effective for producing PCs, yet the substantial consumption of activating agents leads to elevated wastewater treatment costs, thereby confining its use mainly to high-value electrode materials. Emerging technologies, including FJH, offer advantages in terms of lower energy consumption, but their large-scale implementation remains challenging due to high equipment costs and limited scalability.
(6) However, the transformation of waste plastics into high-value energy storage products, such as components for batteries and SCs, holds considerable promise for resource recovery. However, scaling up these processes encounters multiple challenges across technological, regulatory, and societal dimensions. Technologically, the chemical inertness of plastics hampers efficient degradation, and energy storage applications require materials of exceptional purity. Current recycling methods, including pyrolysis and catalytic conversion, are often energy-intensive and yield complex mixtures of products, limiting their industrial viability. For example, the thermal treatment of chlorinated polymers such as PVC may generate dioxins, which can contaminate the resulting C materials. Furthermore, various plastic additives (e.g., flame retardants and plasticizers) may adversely affect the electrochemical behavior of electrodes, necessitating the development of targeted pretreatment strategies. In addition, the lack of a unified global classification system for plastic waste highlights deficiencies in policy frameworks and standardization. To achieve efficient energy recovery from waste plastic, it is essential to clearly define its classification as a “resource.” Without such recognition, certain plastics, especially electronic polymers containing heavy metals, may be treated as hazardous waste, which can impose restrictions on cross-border transport. Furthermore, the absence of standardized LCA frameworks for plastic-derived energy storage materials may result in controversies related to secondary environmental impacts. From a societal and commercial perspective, public confidence remains a concern. Public acceptance of “plastic waste-derived batteries” may be hindered by concerns regarding safety, particularly the risks of electrolyte leakage or thermal runaway. Furthermore, recycled plastic feedstocks generally involve higher supply chain costs than virgin materials, making economic viability reliant on policy support, such as C taxation or government incentives. From a systems perspective, the effective transformation of waste plastic to energy storage materials necessitates integration across multiple stages, including waste segregation, advanced sorting techniques (i.e., near-infrared identification), and subsequent chemical processing. Inadequate source separation at the household level, such as mixing PET bottles with multilayer packaging films, can significantly increase downstream sorting and processing costs. Overall, advancing energy storage applications of plastic waste necessitates overcoming interconnected technological, regulatory, and societal barriers. Priority should be given to developing low-emission conversion technologies, establishing internationally harmonized classification standards, and validating economic feasibility through pilot-scale demonstrations.

## Figures and Tables

**Table 1:** Correlation between plastic type, pyrolysis mechanism, and resulting C microstructure.

Plastic Category	Representative Polymers	Key Structural Features	Dominant Pyrolysis Mechanism	Intermediate Species	Resulting C Microstructure	Key Characteristics
Aliphatic polyolefins	PE, PP	Saturated C-C backbone, no functional groups	Random chain scission	Alkanes, alkenes, light hydrocarbons	Amorphous/turbostratic C (low yield)	Low graphitization, poor ordering, limited porosity
O-containing polymers	PET, PMMA	ester/carbonyl groups; PET contains aromatic rings	Depolymerization + fragmentation	Oxygenated compounds, aromatics (for PET)	Partially graphitized C with developed porosity	Moderate ordering, O-induced pore formation
N-containing polymers	PU, polyamide	amine/amide groups	Crosslinking + aromatization	N-containing heterocycles, stable radicals	N-doped C with defect-rich structure	High defect density, enhanced electronic properties, active sites
Halogen-containing polymers	PVC	C-Cl bonds, labile halogen groups	Dehydrochlorination + polyene formation	HCl gas, conjugated polyenes	Porous, defect-rich C	High microporosity, high defect density
Aromatic polymers	PS	Aromatic rings in backbone	Aromatization + condensation	Styrene, polyaromatic intermediates	Relatively highly graphitized C	Higher structural ordering, possible graphitic domains

**Table 3:** Overview of reported waste plastic-based carbons employed as anodes in LIBs and SIBs.

Plastics	Preparation Route	C Material	Electrolyte	Reversible Capacity (mAh g^−1^) [Current Density (A g^−1^)]	Number of Cycles	Ref.
**Lithium-ion batteries**						
Mixed commodity polymers	Sol–gel synthesis and stainless autoclave carbonization.	(HCS/PCF)	1 M LiPF_6_ in EC/DMC/EMC (1:1:1 vol %)	802 [0.5]	500	[139]
		(SnO_2_/HCS/PCF)		1125 [1.0]	400	[140]
PS foam	Urea-assisted carbonization	NPCs		600 [1.0]	200	[138]
PET bottles	Ionothermal pyrolysis	ACs	1 M LiPF_6_ in EC/DMC (1:1 vol %)	460 [0.1]	100	[141]
LDPE	S-assisted hydrothermal activity followed by carbonization	Soft C	1 M LiPF_6_ in EC/DMC/EMC (1:1:1 vol %)	370	200	[142]
HDPE				470		
PE	Thermal oxidation and subsequent catalytic carbonization	Graphite	1 M LiPF_6_ with 5 vol % of FEC additive in EC/EMC (1:1 vol %)	326 [0.2 C]	250	[143]
PP	Microwave-assisted pyrolysis followed by chemical activation using KOH	ACs	1 M LiPF_6_ in EC/DMC (1:1 vol %)	355.1 [0.2]	100	[137]
Waste mask (PP)	Thermal oxidation and subsequent carbonization	Hard C	1 M LiPF_6_ in EC and diethyl carbonate (DEC) (1:1 vol %)	438.1	100	[136]
PE	Two-stage pyrolysis-catalytic conversion process	CNT composites (CNCs)	1 M LiPF_6_ in EC/EMC (3:7 vol %)	522.4	100	[144]
Mixed plastics (PP/PE/PS)	Catalytic carbonization-etching process	Yolk–shell Co_3_O_4_@C	1 M LiPF_6_ in EC/EMC (1:2 vol %)	1066 [0.1]	300	[145]
PE	Microwave-assisted graphene-triggered electromagnetic reaction.	Co-GNP-ZipC	1 M LiPF_6_ in EC/DEC (1:1 vol %)	377	250	[146]
PS		Co-GNP-FmC		509		
**Sodium-ion batteries**						
PS cups	Carbonization under confined reactor	Disordered C	1 M NaClO_4_ in EC/DEC (1:1 vol %)	116	80	[147]
Styrene acrylonitrile (SAN) plastics	Carbonization followed by CO_2_ activation	ACs	1 M NaPF_6_ in EC/DEC (1:1 wt %)	190 [0.003]	100	[148]
Polyolefin plastic wastes	Microwave-triggered pyrolysis in the presence of a Ti_3_AlC_2_ catalyst	CNFs	1 M NaPF_6_ in dimethoxyethane (DME)	142 [0.05]	2000	[149]
Waste tires	Carbonization followed by CO_2_ activation	ACs	1 M NaPF_6_ in EC/DEC (1:1 wt %)	300 [0.01]	20	[150]
	Acid treatment followed by thermal decomposition	Hard C	1 M NaClO_4_ in EC/DEC (1:1 vol %)	203 [0.03]	100	[151]
	Two-stage thermal decomposition	3D vertical graphene	1 M NaClO_4_ in EC/DMC/EMC (1:1:1 vol %) with 2% FEC	252.7 [0.2]	200	[152]
PET	NaCl/KCl-assisted carbonization followed by ball-milling activation	ACs	1 M NaClO_4_ in DMC/EC (1:1 vol %)	217.8 [0.03]	100	[153]
PE	S-assisted thermal decomposition	S-doped carbons	1 M NaClO_4_ in EC/PC (1:1 vol %) with 5 vol % of FEC	662 [0.05]	1	[154]
PP				578 [0.05]		
PS				661 [0.05]		
Waste mask (PP)	Sulfonation followed by carbonization treatment	Hard C	1 M NaClO_4_ in EC/DEC (1:1 vol %)	327.4 [0.1 C]	200	[155]
			1 M NaPF_6_ in DEG/DME	340 [0.01]		[156]
Waste tires	Two-stage pre-oxidation followed by nitridation treatment	N/O co-doped mesoporous hard C	1 M NaPF_6_ in DIGLYME	407 [1.0]	100	[157]
Ester bond-rich waste plastics (PC and PET)	Direct carbonization	Hard C	1 M NaPF_6_ in DMC/EC (1:1 vol %)	327 [0.02]	140	[158]
				342 [0.02]		
PET	Microwave-assisted thermal decomposition		1 M NaClO_4_ in EC/DEC (1:1 vol %)	363 [0.1]	200	[159]
PET	Ethanolamine-mediated aminolysis, co-solvent incorporation, freeze drying, and microwave-induced carbonization	N-doped hard C	1 M NaPF_6_ in DME	452 [0.02]	200	[160]
Floral foam (phenol-formaldehyde foam)	Direct carbonization	Hard C	1 M NaClO_4_ in EC/PC at a 1:1 vol % with 5 wt % FEC	434.9 [0.4]	1000	[161]
Automotive shredder residue (ASR)			1 M NaPF_6_ in EC/DMC (1:1 vol %)	434 [0.01]	100	[162]
Poly(terephthalamide) diamide	Two-stage thermal decomposition		1 M NaPF_6_ in DIGLYME	350 [0.1C]	100	[163]

**Table 4:** Summary of recent studies on waste plastic-derived C products applied as cathode components in LSBs.

Plastics	Preparation Route	C Product	Electrolyte	Reversible Capacity (mAh g^−1^) [Current Rate (C)]	Cycle Number	Ref.
LDPE	MW-enhanced sulfonation coupled with subsequent carbonization	Porous sulfonated C	1 M lithium bis(trifluoromethanesulfonyl)imide (LiTFSI) in the bisolvent of 1,3-dioxolane (DOL) and 1,2-dimethoxyethane (DME) (1:1 at vol %) dissolved with 1 wt % LiNO_3_	776 [0.5C]	200	[186]
PVC	Ball milling followed by KOH- and thiourea-assisted carbonization	N, S co-doped C		836 [1C]	500	[187]
PS foam	Sulfonation treatment followed by H_3_PO_4_-assisted carbonization and S melting infiltration	S, P co-doped C		893 [2C]	800	[188]
PS		N, S co-doped C		1079 [0.1C]	500	[189]
Plastic waste residue	CaCO_3_ NPs-assisted carbonization with H_3_BO_3_	B, N co-doped C	1 M LiTFSI and 0.2 M LiNO_3_ in DME and DOL (1:1 at vol %)	756 [0.5C]	200	[190]
	KOH-assisted Carbonization	N-doped C		623 [0.5C]	200	[191]
Waste mask (PP)	H_2_SO_4_-mediated MW preconditioning followed by intrinsic activation	Porous C	1 M LiTFSI in DOL and DME (1:1 at vol %) with 1 wt % LiNO_3_	1313.6 [0.1C]	400	[192]
	H_2_SO_4_ and urea-mediated MW pretreatment followed by intrinsic activation	S, N and O co-doped C		1459.8 [0.1C]		[193]

## Data Availability

No new data were created or analyzed in this study. Data sharing is not applicable to this article.
